# Supercritical CO_2_‐Induced Porous MXene Films for Electromagnetic Interference Shielding and Infrared Stealth

**DOI:** 10.1002/advs.75974

**Published:** 2026-06-03

**Authors:** Hui Zhao, Shuai Li, Jiabei He, Tong Gao, Jingfeng Wang, Dongxiao Kan, Wangtu Huo, Lixin Chen

**Affiliations:** ^1^ Northwest Institute for Nonferrous Metal Research Xi'an Shaanxi China; ^2^ Shaanxi Key Laboratory of Macromolecular Science and Technology School of Chemistry and Chemical Engineering Northwestern Polytechnical University Xi'an Shaanxi China

**Keywords:** electromagnetic interference shielding, infrared stealth, MXene, phase transition, supercritical CO_2_

## Abstract

Lightweight and flexible porous MXene films have emerged as promising candidates for electromagnetic interference (EMI) shielding and infrared stealth. However, current foaming techniques remain a challenge in preserving the intrinsic properties of MXene while achieving eco‐friendly foaming. Herein, supercritical CO_2_ foaming technology is employed to realize the green foaming of dense MXene films without compromising the inherent structure of the MXene nanosheets. The phase transition of supercritical CO_2_ induces bubble nucleation and growth, effectively unfolding the stacked MXene nanosheets into a porous architecture. This strategy is broadly adaptable and can also be extended to other two‐dimensional (2D) films, such as graphene oxide. Benefiting from highly‐conductive porous skeleton, the foamed porous MXene film exhibits an EMI shielding effectiveness of ∼73.2 dB, which is 21% higher than that of the pristine MXene films. Furthermore, its absolute shielding effectiveness attains 23160 dB·cm^2^/g, surpassing most previously reported MXene‐based porous materials. Notably, the porous MXene films also demonstrate excellent infrared thermal camouflage performance across a broad temperature window. This work not only offers an insight for the foaming of 2D MXene materials but also highlights the considerable engineering prospects of porous MXene films in the field of safety protection.

## Graphical abstract

The phase transition of supercritical CO_2_ induces the foaming of dense MXene film without compromise inherent structure of MXene nanosheet. The foamed porous MXene film manifests remarkable EMI shielding and infrared thermal camouflage performances. Moreover, the foaming strategy is broadly adaptable and can be extended to other two‐dimensional films, such as graphene oxide.

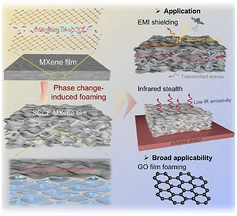

## 1 Introduction

Electromagnetic pollution, recognized as the fourth major public hazard, compromises the reliability of high‐end precision electronic equipment, the security of confidential information, and even threatens human health. With the advent of the artificial intelligence era, the proliferation of high‐frequency, high‐speed, and highly integrated electronic devices further exacerbates electromagnetic radiation issues [1, 2, 3, 4]. Recently, high‐performance electromagnetic interference (EMI) shielding materials have made outstanding contributions in alleviating or eliminating electromagnetic radiation pollution [5, 6]. Nevertheless, the popularity of portable and wearable flexible electronic devices, together with the rapid development of the military field, further requires high‐performance EMI shielding materials characterized by lightweight, thinness, and flexibility, which are unmatched by traditional EMI shielding materials [7, 8, 9, 10]. Moreover, it is also crucial to consider the actual operating environment of electronic equipment. For example, the infrared detection technology could expose high‐precision electronic equipment in the armament field to the risk of being attacked, significantly reducing its battlefield survivability [11, 12]. Therefore, EMI shielding composite films endowed with simultaneous infrared stealth capability can be considered as a promising solution for the next‐generation electronic devices, which are substantial value in both military and civilian fields.

MXene, a new type of 2D transition metal carbide and/or nitride, possesses metallic‐level electrical conductivity and low infrared emissivity (as low as 10% for the mid‐IR wavelengths) [13, 14, 15, 16]. Thus, it is the most promising candidate for fabricating composite films with both EMI shielding and infrared stealth properties. However, due to strong van der Waals forces and hydrogen bonds, MXene nanosheets tend to stack together during the assembling process, resulting in a narrow interlayer space [17, 18, 19]. Such compactness interlayer space not only suppresses the depletion of electromagnetic waves through multiple interfaces inside the material but also imparts a high material density (up to ∼3.8 g/cm^3^) [18]. Porous structure engineering has emerged as an effective strategy to solve the stacking of MXene nanosheets [9, 18, 20, 21], and it has also been proven to be effective in other 2D materials, such as graphene [22] and molybdenum disulfide [23]. The pore structure can introduce numerous free spaces, which cannot only facilitate the multiple reflection and the effective attenuation of electromagnetic waves but also effectively reduce the density of materials, imparting a lightweight characteristic [24, 25]. Furthermore, according to Stefan‐Boltzmann law, the thermal insulation properties of the porous structure are expected to synergize with the intrinsically low infrared emissivity of MXene to boost the infrared stealth performance [26]. These are crucial for aerospace and other advanced fields that require lightweight EMI shielding and infrared stealth materials. At present, porous MXene films are primarily fabricated via two approaches: chemical foaming and physical templating. In the chemical foaming route, small gaseous molecules produced by chemical reactions would aggregate and generate sufficient pressure to overcome the van der Waals force, making the MXene film effectively expand, such as the hydrazine‐induced reduction method [17, 20] and acid‐base neutralization method [18]. Compared with the original MXene film, EMI SE of the as‐fabricated porous MXene films is improved by more than 30%, and they exhibit better infrared stealth performance. Alternatively, the physical template method constructs the three‐dimensional (3D) porous structure of MXene film by removing the low‐stability template. The sacrificial template usually includes organic polymer microspheres (such as polymethyl methacrylate) [21, 27, 28], inorganic microspheres (sulfur) [29], and ice crystal templates [30]. Beyond that, in recent years, it has been reported that 3D printing technology can construct a porous structure from 2D nanosheet materials, achieving superior electrical conductivity and EMI shielding performance [31]. Despite these advances, both foaming and templating strategies suffer from intrinsic defects in the production process, including the use of toxic reducing agents, severe environmental pollution, high‐temperature processing, and stringent equipment demands. Therefore, it is urgent to develop a green foaming technology that can achieve comparable foaming capabilities to the reported complex process, further accelerating the practical application of porous MXene materials.

Supercritical fluid foaming technology, employing supercritical CO_2_ as a physical blowing agent, possesses unique advantages such as non‐toxicity, eco‐friendliness, and excellent foaming ability [32]. This technology can emerge broad development and application prospects in achieving lightweight high‐performance materials. Currently, this foaming technology is primarily aimed at the construction of microcellular structures for polymer materials, including epoxy resin [33] and polymethyl methacrylate [34]. Typically, conductive nanofillers, such as carbon nanotubes, graphene, and MXene, are incorporated into polymers to achieve the purpose of EMI shielding. During the foaming process, these nanofillers were rearranged and oriented along with the cell walls to form a complete conductive network, such as epoxy/graphene composite foam [33] and polyamide/MXene composite foam [35]. These composite foams exhibit superior EMI SE of 30∼80 dB. However, due to the presence of insulated polymers, the fair‐sized thickness (>2 mm) is required to meet the above‐mentioned excellent EMI shielding performance, which affects their integration in the microelectronics industry. Therefore, it is expected to employ supercritical foaming technology to directly foam the MXene film, eliminating the negative impact of insulated polymers. Simultaneously, this foaming technology can circumvent the inherent drawbacks of both foaming and templating strategies. So far, there are few reports on the construction of porous structures in MXene films by supercritical foaming technology, making it difficult to provide theoretical guidance for practical research and application.

Herein, this work proposes supercritical CO_2_ as a foaming agent to construct a lightweight, flexible, self‐supporting MXene film with a microcellular structure. Both theoretical simulations and experimental results have demonstrated that the pressure‐induced phase transition of supercritical CO_2_ can overcome the van der Waals force between the nanosheet interlayers, effectively expanding the dense structure of pristine MXene film and enabling the green production of porous MXene films. The supercritical CO_2_‐foamed MXene (SCCF‐MXene) film manifests a low density (∼0.5 g/cm^3^), remarkable electrical conductivity, flexibility, and outstanding stability in water. Profiting from abundant pore structure and complete conductive network, SCCF‐MXene film exhibits charming EMI shielding performance, which is superior to the original MXene film. In addition, the SCCF‐MXene film also reveals excellent infrared stealth performance. This work laid a substantial theoretical foundation for the future application of high‐performance porous MXene materials.

## 2 Results and Discussion

### 2.1 Fabrication of SCCF‐MXene Films

**FIGURE 1:**
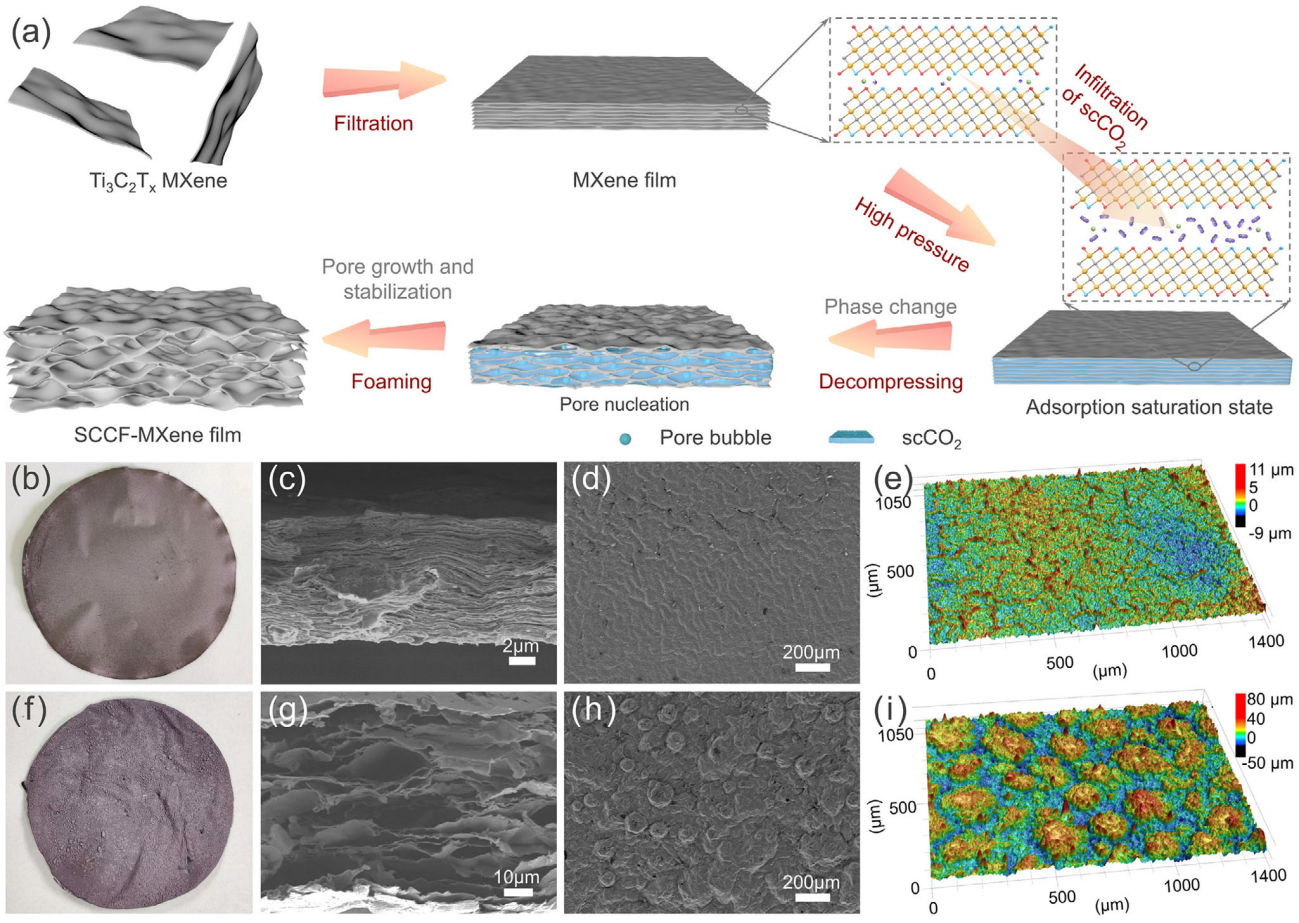
The fabrication and microstructure of the SCCF‐MXene film. (a) Schematic illustration. (b, f) Digital photograph, (c, g) cross‐sectional SEM images, (d, h) superficial SEM images, and (e, i) 3D surface images of (b‐e) MXene film and (f‐i) SCCF‐MXene film.

Microcellular SCCF‐MXene films are constructed via supercritical CO_2_ technology, as shown in Figure 1. Initially, MXene nanosheets, serving as building blocks, are self‐assembled into a compact MXene film via vacuum‐assisted filtration. Subsequently, supercritical CO_2_ fluid is infiltrated into the MXene film and embedded between the interlayers of the nanosheets using a supercritical fluid pump until saturation equilibrium is reached. During this process, the supercritical fluid pump controls the saturated pressure, while an intelligent quick‐opening autoclave controls the saturated temperature. Finally, the pressure is released to disrupt the thermodynamic equilibrium of the system, inducing a phase transition of the supercritical CO_2_. The phase transition would generate bubble nucleation, which would then continue to grow as the pressure drops. The burgeoning CO_2_ bubbles can exert an internal driving force that effectively expands the stacked interlayer structure, thereby transforming the dense MXene film into a 3D microcellular SCCF‐MXene film.

To ensure the outstanding performance of foamed SCCF‐MXene films, the preparation of high‐quality MXene nanosheets is crucial. Monolayer Ti_3_C_2_T*_x_* MXene was prepared via a modified in situ LiF/HCl etching method, as shown in Figure S1. The resulting MXene suspension exhibited a dark green color and a pronounced Tyndall effect, preliminarily indicating that Ti_3_C_2_T*_x_* MXene nanosheets were successfully synthesized and possessed superior dispersion in water. From the high‐resolution TEM image (Figure S2a), the Ti_3_C_2_T*_x_* MXene nanosheets exhibit a monolayer structure with a transverse size of 1.2 µm. Importantly, these nanosheets exhibit clear edges and no impurities on the surface, indicating that the Ti_3_C_2_T*_x_* MXene nanosheets are not oxidized during preparation. Meanwhile, the AFM image (Figure S2b) indicates that the thickness of Ti_3_C_2_T*_x_* MXene nanosheets is approximately 1.5 nm, slightly exceeding the theoretical thickness of 1 nm. The phenomenon is consistent with previously reported results and is attributed to factors such as the substrate in the actual test [36]. These results manifest that Ti_3_C_2_T*_x_* MXene with a monolayer structure was successfully exfoliated. Furthermore, the XRD patterns also occur in a significant structural evolution (Figure S2c). The (104) diffraction peak (near 2θ = 39°) of the MAX phase disappears, and the (002) diffraction peak shifts from 9.6° to 6.6°, indicating that the Al layer is completely etched and the spacing between the nanosheets expands. In conclusion, the above results substantiate that high‐quality Ti_3_C_2_T*_x_* MXene nanosheets are successfully synthesized, laying a robust foundation for the construction of SCCF‐MXene films.

The as‐prepared MXene nanosheet dispersion was assembled into an independent self‐supporting MXene film through vacuum‐assisted filtration. As illustrated in Figure 1b, the assembled Ti_3_C_2_T*_x_* MXene film displayed typical lavender metallic luster, and from the cross‐sectional SEM (Figure 1c), the MXene nanosheets are stacked in parallel to form a dense interlayer stacking structure, which is a prototypical micromorphology of MXene film [2, 36]. Also, this is the fundamental reason that hinders further improvement in the EMI shielding performance of the MXene film. In comparison, the supercritical CO_2_‐foamed SCCF‐MXene film retains the lavender color of the original MXene film, however, its surface grows into rugged (Figure 1f). Furthermore, SCCF‐MXene film develops a continuous pore structure, with pore sizes ranging from tens of nanometers to several micrometers between the parallel layers, accompanied by an increased thickness to 66.5 µm (Figure 1g). These demonstrate that porous architecture in the SCCF‐MXene film has been successfully constructed by supercritical CO_2_. Mercury intrusion porosimetry was measured to investigate the pore size distribution and porosity of pristine MXene film and SCCF‐MXene film (Figure S3, Table S1). The pristine MXene film has a porosity of merely 2.8%, corresponding to its compact packed structure. Whereas, the SCCF‐MXene film exhibits a porosity of 88.2% with a pore size distribution ranging from tens of nanometers to several micrometers, unequivocally evidencing effective foaming. In addition, compared with the smooth dense MXene film (Figure 1d, Figure S4a), the surface of SCCF‐MXene film manifests pronounced bulges (Figure 1h, Figure S4b), originating from the outward expansion of the gas triggered by the phase transition of supercritical CO_2_. Correspondingly, the surface roughness of the SCCF‐MXene film reaches 10.6 µm (Figure 1i), significantly higher than that of the pristine MXene film (Ra = 1.5 µm, Figure 1e). 3D surface reconstructions are consistent with the macroscopic optical observations. The above results evince that the supercritical CO_2_ foaming method can effectively construct the SCCF‐MXene film with a rich porous structure. This rich porous structure, along with the interconnectedness between the nanosheets, imparts excellent flexibility to the SCCF‐MXene film (Figure S5) and reasonable mechanical properties (Figure S6), allowing it to be bent and wrapped around a glass rod without any cracks.

### 2.2 Supercritical CO_2_ Foaming Mechanism of SCCF‐MXene Films

**FIGURE 2:**
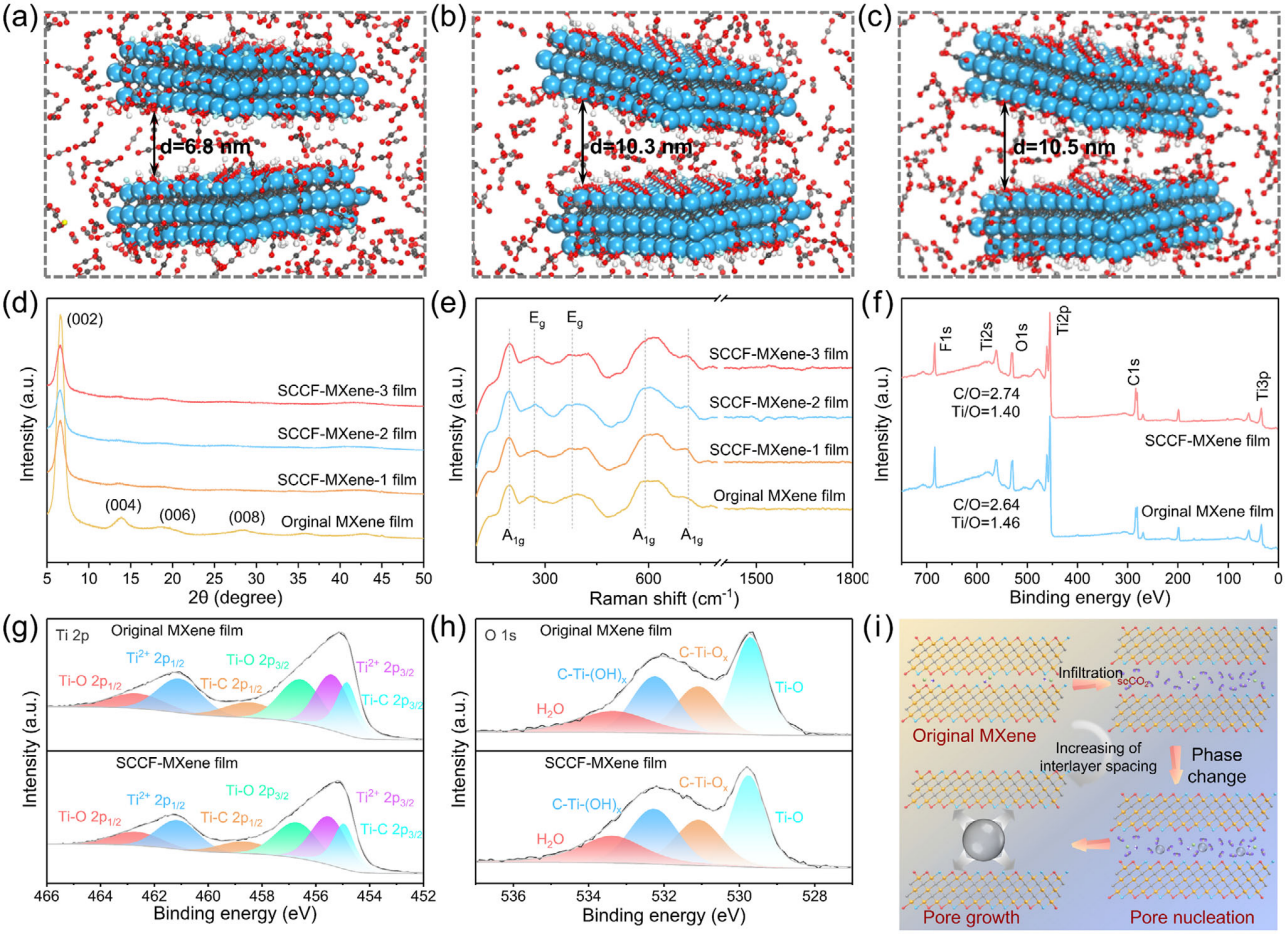
Supercritical CO_2_ foaming process of MXene film. (a‐c) Molecular dynamics simulations illustrating the adsorption and diffusion of CO_2_ inside MXene interlayer at (a) 8 MPa, (b) 15 MPa, and (c) 22 MPa. (d) XRD patterns, (e) Raman, (f) XPS survey spectra, high‐resolution Ti 2p (g) and O1s (h) spectra of the MXene film and SCCF‐MXene film. (i) Supercritical CO_2_ foaming mechanism.

To elucidate the supercritical CO_2_ foaming mechanism of MXene films, the mass transfer process of supercritical CO_2_ under high pressure and the foaming process upon pressure release were explored. During depressurization, cell nucleation and growth are accomplished, however, the adsorption and distribution of supercritical CO_2_ inside the MXene film before pressure release play a decisive role in nucleation and growth. Directly, real‐time monitoring of CO_2_ adsorption and diffusion under high pressure is extremely difficult under actual experimental conditions. Furthermore, the rapid depressurization characteristic of supercritical CO_2_ makes it difficult to accurately determine the adsorption of supercritical CO_2_ using gravimetric methods. Therefore, it is challenging to precisely quantify the phase transition, nucleation, and pore evolution processes. To address the limitations, molecular dynamics were employed to investigate the adsorption behavior of supercritical CO_2_ in MXene interlayers, as shown in Figure 2a–c and Figure S7. Upon pressurization, gaseous CO_2_ can be evolved into supercritical state, which enables more CO_2_ molecules to penetrate into the interlayers of the MXene film. Moreover, with the infiltration of CO_2_, the interlayer spacing between the MXene nanosheets expands, facilitating additional uptake in the saturated state. This process ensures that enough CO_2_ gas escapes to overcome van der Waals forces between adjacent nanosheets, thereby establishing a porous framework. Furthermore, the adsorption of supercritical CO_2_ under varying saturation pressures was also simulated. As the pressure increases from 8 to 15 MPa, the number of intercalated CO_2_ molecules and the corresponding interlayer spacing of the MXene nanosheets synchronously augment. Beyond 15 MPa, however, when the pressure continuously increased to 22 MPa, the interlayer spacing and the number of CO_2_ molecules remained almost unchanged (Figure 2b,c). The above result indicates that the adsorption saturation equilibrium of supercritical CO_2_ is effectively attained at 15 MPa inside the MXene film.

Furthermore, the gas nucleation and subsequent pore evolution during the foaming process were regulated via varying the mass‐transfer pressure, thereby controlling the ultimate pore morphology and indirectly elucidating the adsorption of supercritical CO_2_. As shown in Figure 1g and Figure S8, when the saturation pressure increases to 15 MPa, the pore structure of the SCCF‐MXene film matures into a more plentiful state, accompanied by a substantial increase in thickness from 9 µm (pristine MXene film) to 66.5 µm. This structural expansion results in a pronounced decline in the density, from 3.8 g/cm^3^ in pristine MXene film to 0.5 g/cm^3^ in the porous SCCF‐MXene film (Figure S9). Similarly, the pore structure, thickness, and density of the SCCF‐MXene‐3 film at 22 MPa remain essentially invariant. This behavior can be explained by classical nucleation theory [37]. The low supercritical CO_2_ concentration in the MXene film augments the energy barrier required for gas nucleation, thereby suppressing nucleation density and constraining stacked nanosheet expansion. Conversely, the enhanced supercritical CO_2_ uptake promotes more nucleation, resulting in a porous MXene film with a profuse pore structure. However, once supercritical CO_2_ adsorption reaches saturation in the MXene film, further augment in pressure cannot significantly alter the nucleation and growth of pores. These microstructures of various SCCF‐MXene films are consistent with the molecular dynamics results, indicating that supercritical CO_2_ can penetrate into the MXene film and reach a saturated state at 15 MPa. Such adsorption saturation in the MXene film ensures that the supercritical CO_2_ phase transition can drive the construction of 3D porous structure.

The evolution of chemical composition and structure for MXene films during the foaming process was analyzed by XRD, Raman, and XPS. In the XRD patterns (Figure 2d), the intrinsic (002) peak (2θ = 6.6°) for MXene is discernible in the MXene film and the three SCCF‐MXene films. However, compared with the unfoamed MXene film, the (002) peak intensities of the porous SCCF‐MXene films are markedly attenuated, and their half‐peak widths are broadened. Moreover, the high‐position peaks, such as (004), (006), and (008), have essentially vanished. These changes in characteristic peaks are attributed to the suppressed stacking tendency of the nanosheet and enhanced disorder of the film [38], indicating that the MXene film has been successfully foamed. Notably, the (002) peak intensities of SCCF‐MXene‐2 and SCCF‐MXene‐3 films are nearly identical, however, compared with SCCF‐MXene‐1 film, their (002) peak intensities are weaker. This phenomenon also indirectly indicates that the mass‐transfer pressure of 15 MPa is sufficient to drive extensive penetration of supercritical CO_2_ into MXene film and enable full foaming, which is consistent with the simulated results. Importantly, there is no new TiO_2_ peak in the porous SCCF‐MXene film, indicating that the oxidation of MXene cannot occur during solution processing or supercritical foaming. Figure 2e shows the Raman spectra of the MXene film and SCCF‐MXene films. The typical A_lg_ (Ti, C) vibration mode (200, 590, 716 cm^−1^) and E_g_ (Ti, C) vibration mode (270, 378 cm^−1^) in the pristine MXene film are well preserved in the SCCF‐MXene film [39, 40]. And no TiO_2_ peaks or free carbon D and G bands are observed, indicating that the porous SCCF‐MXene film has not been oxidized or degraded. Furthermore, XPS analysis was measured to investigate the chemical composition of the MXene films before and after foaming, as shown in Figure 2f–h. Specifically, the Ti/O atomic ratio (1.46) and C/O atomic ratio (2.64) in the pristine MXene film are nearly identical to those in the foamed SCCF‐MXene film (Figure 2f). Likewise, the signal peaks intensities and areas of the high‐resolution Ti 2p and O 1s spectra for the pristine MXene film are also congruent to those of the porous SCCF‐MXene film (Figure 2g,h). Collectively, these results indicate that the supercritical foaming process cannot remodel the chemical composition and structure of the MXene nanosheets, instead, it merely reconfigures the stacked architecture into an interconnected 3D porous framework.

Based on the above analysis, the foaming mechanism induced by supercritical CO_2_ can be summarized as an “adsorption diffusion−phase transformation nucleation−bubble growth” process, as shown in Figure 2i. Due to the presence of intercalated H_2_O and Li^+^ in the nanosheets interlayer, supercritical CO_2_ fluid can penetrate into the vacuum‐filtered MXene film and further expand the interlayer spacing, allowing more supercritical CO_2_ to be adsorbed in MXene film under saturation state. Upon pressure release, the confined supercritical CO_2_ undergoes a phase transition within MXene film. During this transition, supercritical CO_2_ nucleates to form small bubbles that subsequently grow with the further drop of pressure. However, the sealed MXene film allows only a portion of CO_2_ to escape through the edges or the tiny channels left after filtration. Numerous maturing CO_2_ bubbles are trapped inside the MXene film, generating high internal pressure and exerting strong outbound forces perpendicular to the MXene film plane. This offsets the van der Waals forces between adjacent nanosheets, facilitating the expansion of the stacked MXene nanosheets. Ultimately, a cavity‐rich, 3D porous architecture emerges within the initially dense MXene film. Importantly, the supercritical CO_2_ foaming conditions are mild, and the phase transition of CO_2_ retains both the chemical structure and unparalleled inherent properties of MXene, providing a robust underpinning for EMI shielding and infrared stealth. Furthermore, CO_2_ is widely available and can be recycled and reused after depressurization. Compared with previously reported methods, the supercritical foaming process proceeds without chemical reactions, avoiding the issues of residual byproducts, toxic reducing agents, and high‐temperature thermal reduction. This approach advances the green and sustainable development of 3D porous MXene materials. More critically, the essence of this technology lies in harnessing the phase transition of CO_2_, thereby offering broad applicability to other classes of 2D materials. For instance, employing graphene oxide (GO) as a raw material, 3D porous GO film (Figure S10) can also be fabricated via a combination of vacuum‐assisted filtration and supercritical CO_2_ foaming.

In addition, the superior durability and stability of SCCF‐MXene films in water should also be underscored. While the surface of the MXene nanosheets in the SCCF‐MXene film still retains ample hydrophilic groups, the foaming markedly enhances its surface roughness (Figure 1i), to a certain extent, constructing the micro‐nanostructures that effectively hinder the spreading behavior of droplets. Consequently, compared with the excellent hydrophilicity of pristine MXene film (contact angle of 55°), the contact angle of SCCF‐MXene film reaches 82° (Figure S11). Furthermore, the aqueous stability of both films under sonication was assessed (Figure S12). Stemming from its excellent hydrophilicity, the pristine MXene film readily wets and softens in water, and undergoes rapid structural degradation, collapsing completely within 300 s of sonication (Figure S12, Video S1). However, SCCF‐MXene film, characterized by reduced wettability and low density, can maintain structural integrity even after 300 s of ultrasound treatment (Figure S12, Video S1). Therefore, the supercritical foaming strategy can effectively suppress the water wettability of MXene‐based films, ensuring their operational reliability in EMI shielding and infrared stealth applications in high humidity environments.

### 2.3 EMI Shielding Performances of SCCF‐MXene Films

**FIGURE 3:**
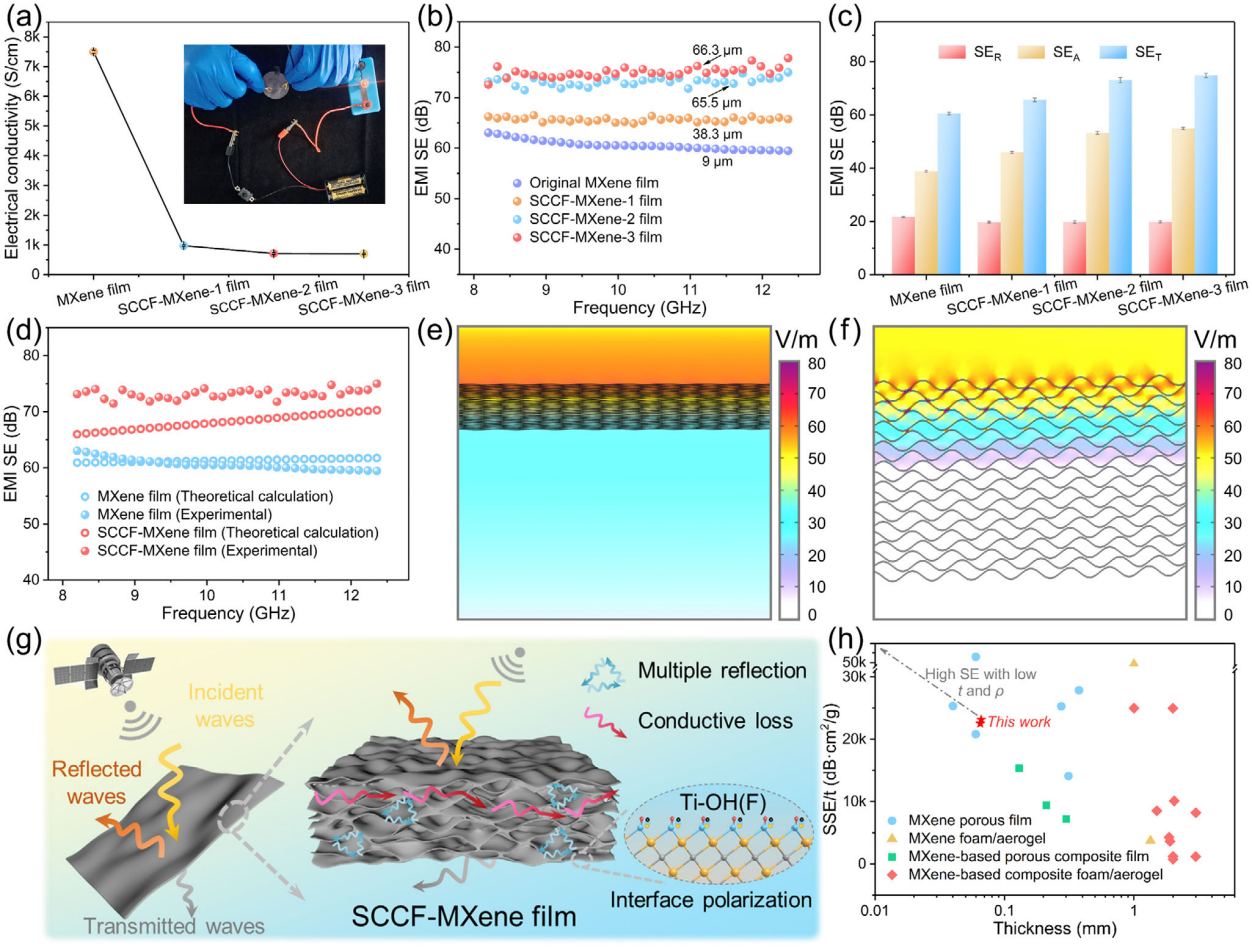
Electrical conductivities and EMI shielding performances of SCCF‐MXene films. (a) Electrical conductivities, (b) EMI SE, and (c) SE_R_ and SE_A_ of the MXene film and SCCF‐MXene film. (d) Experimental and calculated EMI SE of the MXene film and SCCF‐MXene‐2 film. Simulation of the electric field distribution of (e) MXene film and (f) SCCF‐MXene film. (g) Electromagnetic shielding mechanism of SCCF‐MXene film. (h) Comparison of SSE/*t* and *t* of SCCF‐MXene films with other reported MXene‐based shielding materials.

In non‐magnetic porous materials, a conductive skeleton structure is an indispensable condition for ensuring the attenuation of electromagnetic waves by conduction loss and multiple reflection [41]. As shown in Figure 3a, the electrical conductivity of MXene films and different SCCF‐MXene films was measured. By virtue of the above synthesized high‐quality MXene nanosheets, the pristine MXene film exhibits an electrical conductivity as high as 7500 S/cm. This exceptional conductivity underpins the superior EMI shielding effectiveness (SE) of MXene films, while simultaneously giving rise to pronounced interface reflection [42]. However, after foaming, the electrical conductivity of the SCCF‐MXene films decreases markedly, to 980 S/cm, 710 S/cm, and 700 S/cm for SCCF‐MXene‐1, SCCF‐MXene‐2, and SCCF‐MXene‐3, respectively. This diminution can be ascribed to the introduction of abundant porous architectures, which increase the internal free space within the films. Nevertheless, the electrical conductivity of the SCCF‐MXene films remains above 700 S/cm, far exceeding the critical threshold of 0.01 S/cm for commercial EMI shielding materials [43]. These results indicate that the foamed SCCF‐MXene films preserve an intact 3D conductive porous framework, which facilitates the attenuation of electromagnetic waves in the porous network, thereby endowing the films with outstanding EMI shielding performance.

The EMI shielding performances of SCCF‐MXene films in the X‐band were systematically investigated. Figure 3b exhibits the EMI SE of pristine MXene and various SCCF‐MXene films. Pristine MXene film with 9 µm exhibited an EMI SE of 60.6 dB, which is consistent with previously reported results [2]. In contrast, while the electrical conductivity of the foamed SCCF‐MXene films decreases, the abundant pore structures could induce extra multiple internal reflections/scatterings of electromagnetic waves inside the film, thereby yielding satisfactory shielding performance. Notably, the average total EMI SE of the SCCF‐MXene‐2 film is as high as 73.2 dB, significantly surpassing the requirements for commercial EMI shielding materials [44]. Moreover, the SCCF‐MXene‐3 film, possessing comparable electrical conductivity and pore architecture, can also exhibit such remarkable EMI shielding performance. This observation, together with the electrical conductivity results, further corroborates that the MXene film has reached adsorption saturation and equilibrium at 15 MPa, and further increasing the saturation pressure exerts little influence on the porous structure of SCCF‐MXene films. Simultaneously, the average reflection shielding effectiveness (SE_R_) and average absorption shielding effectiveness (SE_A_) were calculated according to Equations (S4–S6), as illustrated in Figure 3c. The SE_R_ is governed by the impedance matching at the interface between the film and the air [45, 46]. According to the impedance matching theory, higher electrical conductivity results in a larger SE_R_. Nevertheless, the total EMI SE exhibits an opposite trend with SE_R_ value: SCCF‐MXene‐3≈SCCF‐MXene‐2>SCCF‐MXene‐1>pristine MXene film. This indicates that the difference in EMI SE between MXene film and SCCF‐MXene films primarily originates from SE_A_. For SCCF‐MXene films, the attenuated electrical conductivity suppresses the motion of free electrons under an alternating electric field, thereby diminishing conduction loss and leading to a decrease in SE_A_. However, from structural characterizations, SCCF‐MXene films exhibit a well‐interconnected 3D porous architecture formed by MXene nanosheets, while the MXene nanosheets retain their intrinsic abundant terminal groups during the foaming process. Compared with pristine MXene film, electromagnetic waves can undergo multiple reflections in the internal porous network of SCCF‐MXene films. Meanwhile, these multiple reflections/scatterings could also enhance the interactions between the waves and the surface functional groups, intensifying polarization relaxation loss [47]. More importantly, the increased thickness imparted by the foaming prolongs the propagation path of electromagnetic waves, thereby enhancing both multiple reflection and dipolar polarization losses and ultimately intensifying electromagnetic absorption. Consequently, the gain of SE_A_ arising from the porous architecture completely outweighs the reduction in SE_A_ caused by decreased conductivity. In other words, the multiple interfacial reflections/scatterings within the porous framework contribute dominantly to electromagnetic attenuation, and the EMI SE is further elevated as the pore structure increases.

Furthermore, the theoretical EMI SE of non‐magnetic films was estimated using Simon formalism (Equation 7, Supporting Information), as illustrated in Figure 3d [2, 17]. For the dense MXene film, the EMI SE obtained from VAN measurements is in close agreement with the values predicted by Simon formalism. In contrast, the calculated EMI SE of the porous SCCF‐MXene film shows a pronounced deviation from the experimental data. This further corroborates that multiple reflections/scatterings induced by the porous architecture increase the absorption loss of electromagnetic wave, which in turn promotes the EMI shielding performance of the porous SCCF‐MXene film. Furthermore, finite element simulations (COMSOL, Figure S13) were conducted to elucidate the attenuation behavior of electromagnetic waves in the SCCF‐MXene films under the far‐field radiation mode. Figure 3e,f present the simulated electric field distributions in the pristine MXene and SCCF‐MXene films. In the MXene film (Figure 3e), the high electrical conductivity and severe impedance mismatch result in a strong electric field intensity above the dense stacking structure, indicating that most incident electromagnetic waves are reflected. In contrast, after introducing porous structure, the impedance mismatch is effectively mitigated, and the electric field intensity at the top region of the film is markedly alleviated (Figure 3f), signifying lower reflection and enhanced penetration of electromagnetic waves into the film. Meanwhile, the electric field strength inside the SCCF‐MXene film decreases significantly, implying substantial attenuation of electromagnetic waves within the material. The COMSOL simulation results are consistent with both the theoretical calculations and experimental measurements, further confirming that the enhanced electromagnetic absorption from porous structures is the primary reason for the improved EMI shielding performance of the SCCF‐MXene films. Collectively, the incorporation of multiple reflections/scatterings, coupled with the intrinsic conduction loss induced by the MXene porous framework and dipolar polarization loss associated with surface terminal groups, synergistically amplifies electromagnetic wave attenuation, thus significantly advancing the EMI shielding performance of porous SCCF‐MXene films. Moreover, the conversion of electromagnetic waves is investigated using the power coefficients (R, A, and T) from the point of energy, as illustrated in Figure S14. The R value exceeds the A value for the SCCF‐MXene films, indicating that the dissipation of electromagnetic waves predominantly occurs through reflection‐adsorption but reflection‐dominant shielding mechanism. The variation trends of R and A are consistent with the results of SE_R_ and SE_A_. Owing to multiple reflections/scatterings, coupled with strong dipolar polarization relaxations on the nanosheet surfaces functional groups, the R value of the SCCF‐MXene film decreases to 0.98, which is lower than that of various MXene‐based EMI shielding films (>0.99).

In general, the thickness is a crucial parameter governing the EMI shielding performance of materials [48]. By tuning the mass of MXene nanosheets, the thickness of SCCF‐MXene films can be precisely modulated. The EMI shielding performance of SCCF‐MXene films at various thicknesses is presented in Figure S15. Given the strong dependence of SE_A_ on thickness, the SCCF‐MXene film can achieve EMI SE exceeding 80 dB with increasing thickness. Remarkably, even at a mass of only 30 mg, the SCCF‐MXene film exhibits an EMI SE of 59.1 dB, comparable to that of the pristine MXene film with 45 mg. These results highlight that the introduction of porous architecture enables the realization of comparable EMI shielding performance at reduced material consumption, facilitating the industrial application of MXene‐based EMI shielding materials.

Based on the above analysis, the EMI shielding mechanism of the porous SCCF‐MXene films is systematically illustrated and summarized in Figure 3g. Unlike the original MXene, the existence of abundant insulated pore structure alleviates the impedance mismatch between the SCCF‐MXene film and air, weakening the reflection of incident electromagnetic waves at the film surface and allowing a larger portion to penetrate into the film interior. The penetrated electromagnetic waves would be attenuated through the following three modes. First, the continuous MXene porous skeleton can generate the induced microcurrents under the electromagnetic field, dissipating electromagnetic energy as heat through conduction loss. Then, the original functional groups retained on the MXene surface can provide numerous dipole centers, generating dipole polarization relaxation under electromagnetic fields, thus introducing significant dipole polarization loss. Furthermore, the abundant interfaces in the porous structure can promote multiple reflections/scatterings of electromagnetic waves. The multiple reflections/scattering can effectively prolong their propagation path and enhance interaction numbers onto the MXene skeleton and dipole centers, further converting and depleting electromagnetic waves. In conclusion, the integration of several loss mechanisms endows the porous SCCF‐MXene films with remarkable EMI shielding performance, highlighting their potential applications as lightweight EMI shielding materials in aerospace and other fields.

Beyond outstanding EMI shielding performance, the thickness (*t*) and density (*ρ*) of the materials are also critical evaluation parameters for practical applications in aerospace and wearable electronics. A more comprehensive and practical metric, the specific SE/*t* (SSE/t), provides valuable insight into evaluating the EMI shielding capability of materials [49]. The specific SE (SSE) is defined as the ratio of EMI SE to *ρ*. Accordingly, SCCF‐MXene‐2 and SCCF‐MXene‐3 films exhibit nearly identical SSE/t values, significantly outperforming the original MXene film (Figure S16). Further, we compared the SSE/*t* and thickness of the SCCF‐MXene films with other MXene‐based shielding materials to assess the superiority of the film (Figure 3h and Table S2). As a result, SCCF‐MXene films exhibit both remarkably high SSE/t (23160 dB·cm^2^/g) and low thickness (66 µm), outperforming most reported MXene‐based EMI shielding materials. Although MXene foams induced by hydrazine reduction reaction and porous N‐MXene films induced by acid‐base neutralization reaction achieve higher SSE/*t* values at comparable thicknesses, both foaming strategies inherently involve chemical reactions, leading to unavoidable issues, such as residual byproducts and pollution from toxic reducing agents [17, 18]. Such drawbacks hinder the advancement of 3D porous MXene materials toward green, sustainable processing. By contrast, supercritical foaming technology offers an environmentally benign route, while the SCCF‐MXene films exhibit satisfied EMI shielding performance. In addition, SCCF‐MXene film can possess almost identical EMI shielding ability under water‐oxygen physical attack, with only a ∼3% reduction after ultrasound treatment of 1 h (Figure S17). More importantly, the SCCF‐MXene film presents outstanding environmental stability. After storing at the ambient conditions and 75%RH at room temperature for 30 days, the EMI SE degraded by only 5.1% and 8.9%, respectively (Figure S18). The above practical characteristics would offer promising potential for applications in aerospace, wearable, and microelectronic devices.

### 2.4 Infrared Stealth Performances of SCCF‐MXene Films

**FIGURE 4:**
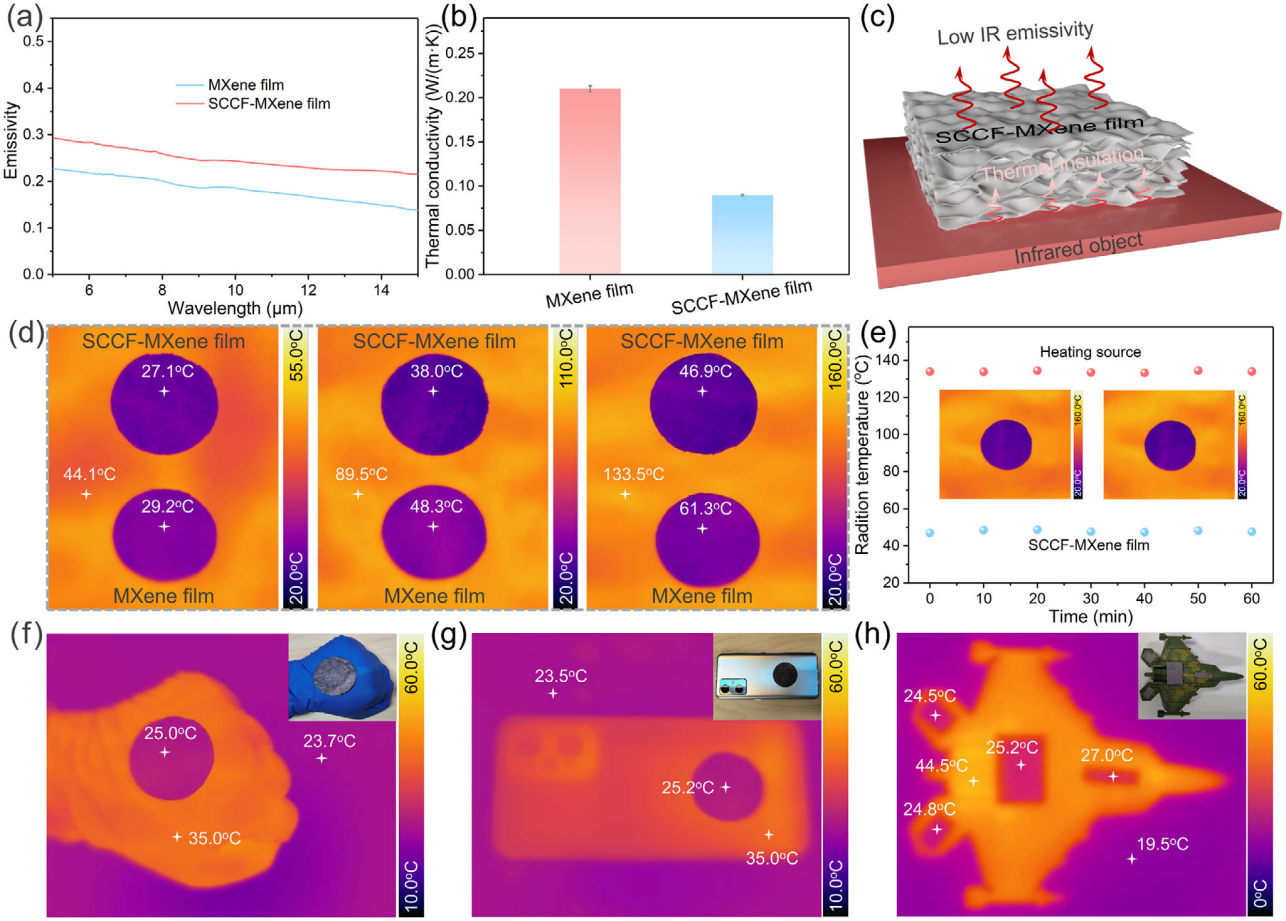
Infrared stealth performance of SCCF‐MXene film. (a) Infrared emissivity and (b) thermal conductivity of MXene film and SCCF‐MXene film. (c) Infrared stealth characteristic of SCCF‐MXene film. (d) Infrared images of MXene film and SCCF‐MXene film at different object temperatures of 45°C, 90°C, and 135°C. (e) Long‐term infrared stealth performances of the SCCF‐MXene film at different object temperatures of 135°C. Infrared stealth effect of (f) skin, (g) mobile phone, and (h) military aircraft model covered by SCCF‐MXene film.

To counter the increasingly sophisticated infrared (IR) detection technologies employed in modern military applications, weapons, transport vehicles, and even personnel need to be camouflaged across diverse thermal environments to ensure seamless integration with their surroundings [44, 50, 51]. Therefore, the development of infrared stealth materials is an effective strategy to regulate thermal signatures. According to the Stefan‐Boltzmann law, IR stealth can be realized by lowering the IR emissivity. MXene possesses an intrinsically low mid‐infrared emissivity (0.20 at 5–15 µm, Figure 4a), much lower than other 2D materials, such as graphene and graphene oxide (GO) [52, 53, 54]. Importantly, the as‐prepared porous SCCF‐MXene film retains the unparalleled inherent properties of MXene, maintaining a low emissivity (∼0.25) comparable to that of the original MXene film, which ensures its excellent inherent infrared stealth capability. In addition, reducing the surface temperature of protected targets provides another pathway for IR camouflage, which is typically achieved through the thermal insulated porous materials [35, 55]. The as‐prepared porous SCCF‐MXene film also integrates the porous thermal insulated structure with the through‐plane thermal conductivity of only 0.09 W/(m·K) (Figure 4b). This depressed thermal conductivity suppresses the diffusion of heat from high‐temperature targets, contributing to infrared thermal camouflage. Compared with dense MXene film in this work and previously reported MXene‐based films, SCCF‐MXene film exhibits superior thermal insulation performance in the direction perpendicular to the plane (Table S3). Therefore, the synergy between the low IR emissivity of MXene and the thermal insulation imparted by 3D porous architecture enables SCCF‐MXene film to achieve outstanding IR thermal camouflage performance (Figure 4c).

To evaluate the thermal camouflage performance of the porous SCCF‐MXene film, heating stages with different temperatures were employed to simulate targets under various environmental conditions. The films were placed on the heating stage, and their time‐dependent radiative temperatures, along with that of the targets, were recorded using an IR thermal imager. Figure 4d presents the IR images of pristine MXene film and SCCF‐MXene film at various stage temperatures. With increasing heating stage temperature, the radiative temperatures of both MXene and SCCF‐MXene films remain markedly lower than that of the heating stage. Notably, compared with pristine MXene film, the porous SCCF‐MXene film exhibits substantially lower radiative temperatures. For instance, when the heating stage is set at 135°C, the surface temperature of the SCCF‐MXene film is only 46.9°C with a reduction of 88.1°C. Even, the SCCF‐MXene film appears nearly indistinguishable from room temperature at a stage temperature of 45°C. These observations unequivocally demonstrate that the SCCF‐MXene film possesses superior infrared stealth capability. Furthermore, the long‐term stability for the IR stealth performance of the SCCF‐MXene film was assessed by continuous operation on heating stages of 45°C, 90°C, and 135°C (Figure 4e and Figure S19). The SCCF‐MXene film can maintain stable surface temperatures of approximately 27°C, 38°C, and 47.5°C over 60 min, respectively, indicating their remarkable thermal camouflage reliability. The outstanding IR stealth capability of the SCCF‐MXene film renders it promising candidate for diverse practical applications. As illustrated in Figure 4f,g, the SCCF‐MXene film exhibits a radiation temperature nearly indistinguishable from the ambient environment, whether applied in the surface of human skin or consumer electronic products, demonstrating excellent camouflage adaptability across different target bodies. Beyond medium‐ and high‐temperature thermal camouflage, the SCCF‐MXene film also exhibits remarkable thermal camouflage capability at low temperatures. As illustrated in Figure S20, the SCCF‐MXene film was placed on an ice block under frozen conditions to measure its low‐temperature thermal camouflage. The SCCF‐MXene film exhibits a similar temperature as the environment, making it promising for application in complex and harsh environments, while also demonstrating its ability for thermal camouflage in a wide temperature range. Moreover, the SCCF‐MXene film can effectively reduce the apparent temperature released by the military aircraft model to the surrounding temperature (Figure 4h), demonstrating its substantial promise for aerospace infrared stealth applications. In conclusion, the dual functionalities of EMI shielding and IR camouflage endow the SCCF‐MXene films with significant prospects for security protection in both military or civilian fields.

## 3 Conclusion

In summary, we have successfully developed porous MXene film by supercritical CO_2_ foaming technology, in which the phase transition of supercritical CO_2_ induces the construction of porous architecture. The supercritical foaming technology is environmentally benign and cannot compromise the inherent properties of the MXene. Also, this method is highly versatile and can be extended to the foaming of other 2D material films. Owing to the intact porous skeleton, the foamed MXene films exhibit a low density of 0.5 g/cm^3^, while the EMI SE and absolute SE can reach 73.2 dB and 23160 dB·cm^2^/g, respectively. Notably, the porous MXene films also demonstrate excellent infrared thermal camouflage performance over a broad temperature range. The lightweight and flexible SCCF‐MXene film with outstanding EMI shielding and IR stealth performance holds significant engineering potential for security protection applications in both military or civilian fields.

## 4 Experimental Section/Methods

### 4.1 Materials

Ti_3_AlC_2_ powder (≥98.0%, 400 mesh) was purchased from 11 Technology Co. Ltd., China. Lithium fluoride (LiF, AR) and Hydrochloric acid (HCl, 9 mol/L) were obtained from Aladdin Reagent Co. Ltd., China. Graphene oxide (GO) was procured from Shanghai Yuanye Bio‐Technology Co. Ltd., China. All chemical reagents were directly used without further purification.

### 4.2 Synthesis of Ti_3_C_2_T_x_ MXene

Ti_3_C_2_T*_x_* MXene was prepared by modified LiF/HCl etching method. First, LiF (2 g) was dissolved in 9 M HCl solution (40 mL) and then stirred to form the etching solution in Teflon vessel. Then, Ti_3_AlC_2_ powder (2 g) was slowly added into the etching solution and stirred at 40°C for 40 h. Afterward, the obtained suspension was repeatedly centrifuged at 3500 rpm with deionized water until reaching a pH value of 6, and the lower Ti_3_C_2_T*_x_* MXene sediment was collected, this is multilayered Ti_3_C_2_T*_x_* slurry. Finally, the Ti_3_C_2_T*_x_* MXene slurry was re‐sonicated in deionized water through ice‐bath for 1 h. Following ultrasonication, the suspension was centrifuged at 3500 rpm for 1 h to remove unexfoliated Ti_3_C_2_T*_x_* MXene. The dark‐green supernatant was collected, and for convenient access, the Ti_3_C_2_T*_x_* MXene nanosheet was diluted into 5 mg/mL.

### 4.3 Fabrication of SCCF‐MXene Film

The pristine MXene films were prepared by the vacuum‐assisted filtration method, wherein MXene dispersion was filtered through a cellulose filter membrane (pore diameter 220 nm) to form the self‐standing MXene films. Then, the freestanding MXene films were transferred into a micro‐autoclave, and the supercritical CO_2_ was pumped into the micro‐autoclave through a supercritical fluid pump. The saturated pressure was controlled by the pumping amount of supercritical CO_2_, while the temperature was monitored by an automatic thermometer of the autoclave. The saturated time was set at 1 h to ensure that the MXene film achieved a saturated CO_2_ absorption state. The pressure in the micro‐autoclave was released within 60 s, and the foaming process was carried out simultaneously, and a SCCF‐MXene film was ultimately obtained. The saturated pressures were controlled at 8, 15, and 22MPa, and corresponding SCCF‐MXene films were labeled as SCCF‐MXene‐1, SCCF‐MXene‐2, and SCCF‐MXene‐3, respectively. It should be noted that the SCCF‐MXene film refers specifically to SCCF‐MXene‐MXene‐2 unless otherwise specified.

All characterizations, including materials characterizations and property measurements, and molecular dynamics simulations, are given in the Supporting Information.

## Author Contributions

**Dongxiao Kan**: validation, software, writing – review and editing. **Hui Zhao**: methodology, conceptualization, data curation, formal analysis, investigation, writing – original draft. **Jiabei He**: formal analysis, visualization. **Shuai Li**: formal analysis, visualization, supervision. **Jingfeng Wang**: formal analysis, visualization, validation. **Wangtu Huo**: writing – review and editing, funding acquisition, supervision, project administration, methodology. **Tong Gao**: validation, formal analysis. **Lixin Chen**: methodology, writing – review and editing, supervision.

## Conflicts of Interest

The authors declare no conflicts of interest.

## Supporting information




**Supporting File**: advs75974‐sup‐0001‐SuppMat.docx.

**Supporting Video**: advs75974‐sup‐0002‐VideoS1.mp4.

## Data Availability

The data that support the findings of this study are available from the corresponding author upon reasonable request.
