# Supplementary material for: Supercritical CO2‐Induced Porous MXene Films for Electromagnetic Interference Shielding and Infrared Stealth
Source: Adv Sci (Weinh). 2026 Jun 3:e75974. Online ahead of print. doi: 10.1002/advs.75974 (PMC13336493; doi:10.1002/advs.75974)
Supplement: Supplementary file 1 — Supporting File: advs75974‐sup‐0001‐SuppMat.docx. [file ADVS-9999-e75974-s002.docx]

**Supporting Information**

**Supercritical CO_2_-induced porous MXene films for electromagnetic interference shielding and infrared stealth**

Hui Zhao, Shuai Li, Jiabei He, Tong Gao, Jingfeng Wang, Dongxiao Kan*, Wangtu Huo*, Lixin Chen*

H. Zhao, S. Li, J.B. He, J.F Wang, D.X. Kan, W.T. Huo

Northwest Institute for Nonferrous Metal Research, Xi’an 710016, Shaanxi, China

E-mail: dxkan@nin.com (D.X. Kan); huowt@c-nin.com (W.T. Huo)

T. Gao, L.X. Chen

Shaanxi Key Laboratory of Macromolecular Science and Technology, School of Chemistry and Chemical Engineering, Northwestern Polytechnical University, Xi’an 710072, Shaanxi, China

E-mail: lixin@nwpu.edu.cn (L.X. Chen)

**S1.** **Characterization**

X-ray photoelectron spectroscopy (XPS) of the samples was performed on an ESCALAB250xi XPS system (Thermo Fisher Scientific Co. Ltd., USA). Atomic force microscopy (AFM) was carried out by Dimension Icon equipment (Bruker, German). Scanning electron microscopy (SEM) and transmission electron microscopy (TEM) of the samples were collected on Verios G4 XHR equipment (FEI, USA) and Talos F200X equipment (FEI, USA), respectively. The cross-sections of composite films were prepared by brittlely fracturing under liquid nitrogen for 10 min. The electrical conductivities of the samples were measured by RTS 8 four-probe testing equipment (Guangzhou Four Probes Technology, China). EMI shielding performances of the samples at X-band (8.2-12.4 GHz) were obtained by an MS46322B vector network analyzer instrument (Anritsu Co., Japan) using a waveguide method, with a sample size of 22.86 mm × 10.14 mm. Thermal images of the samples were collected by an infrared thermal (IR) imager of Ti 300 equipment (Fluke, USA) for infrared stealth properties. The infrared emissivity of the samples was obtained on a Nicolet iS50 FTIR spectrometer (Thermo Fisher Scientific, USA) with an integrating sphere. Thermal conductivity of the samples was characterized with Hot Disk TPS2200 thermal constant analyzer (AB Co., Sweden). Mechanical properties of the samples were measured on a SANS CMT8502 universal testing machine (Instron Co., USA) with a loading rate of 1 mm/min at ambient temperature.

**S2.** **Molecular dynamics simulations**

Molecular dynamics (MD) simulations were carried out using the Forcite module in Materials Studio to investigate the adsorption behavior of MXene interlayers in supercritical CO_2_ environment, with a focus on the pressure-related dynamics across four scenarios (0, 8, 15, and 22 MPa). The initial MXene model was constructed based on its intrinsic layered structure and placed within a cubic simulation box containing supercritical CO_2_ molecules. The supercritical CO_2_ was assigned an initial density corresponding to its supercritical state to satisfy the requirements of the target pressure.

The COMPASS II force field was employed to describe interatomic interactions, and geometry optimization was performed until convergence was attained, with an energy threshold of 1×10^-5^ kcal/mol and a force threshold of 0.01 kcal/(mol·Å). Under the NPT ensemble, all simulations were conducted to maintain the target pressures and temperature, with a Nosé-Hoover thermostat and barostat used for regulation. To mitigate edge effects, three-dimensional periodic boundary conditions were applied. The simulations consisted of a 100-ps equilibration stage followed by a 400-ps production stage with a time step of 1 fs, and trajectories were saved every 100 fs for analysis.

**S3. Calculation of EMI shielding effectiveness (SE)**

Scattering parameters (S_11_, S_12_, S_22_, and S_21_) were output by vector network analyzer directly. The power coefficients, reflection coefficients (R), transmission coefficients (T) and absorption coefficients (A), could be evaluated from the S parameters by following formulations:

$R=\left| S_{11} \right|^{2}=\left| S_{22} \right|^{2}$ (Equation 1)

$T=\left| S_{12} \right|^{2}=\left| S_{21} \right|^{2}$ (Equation 2)

$A=1-R-T$ (Equation 3)

EMI SE contained Total (SE_T_), reflection (SE_R_), absorption (SE_A_) could be expressed as following formulations:

${SE}_{T}=-10\log\left( T \right)$ (Equation 4)

${SE}_{R}=-10\log\left( 1-R \right)$ (Equation 5)

${SE}_{A}=-10\log\left( \frac{T}{1-R} \right)$ (Equation 6)

**S4.** **Calculation of theoretical EMI SE by Simon formalism**

$SE=50+10\log\frac{\sigma}{f}+1.7t\sqrt{\sigma f}$ (Equation 7)

**S5. Supplementary Figures**


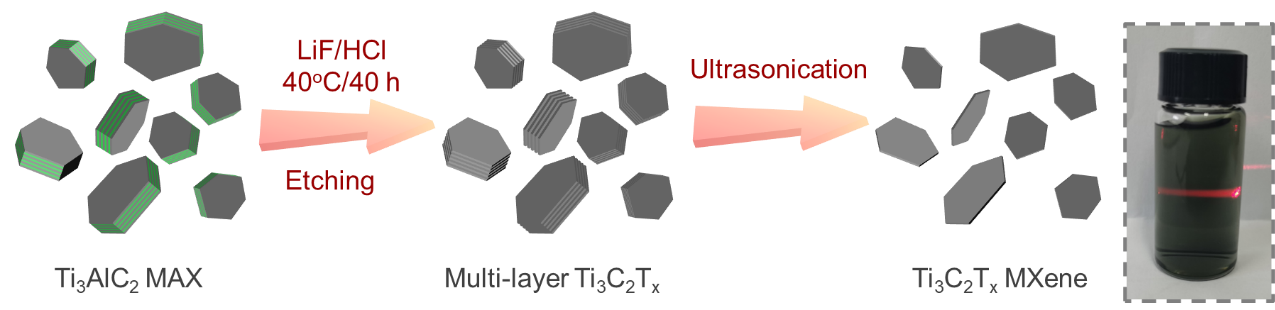


**Figure S1** Schematic illustration for the fabrication process of monolayer Ti_3_C_2_T_x_ MXene and the optical photograph of MXene suspension.


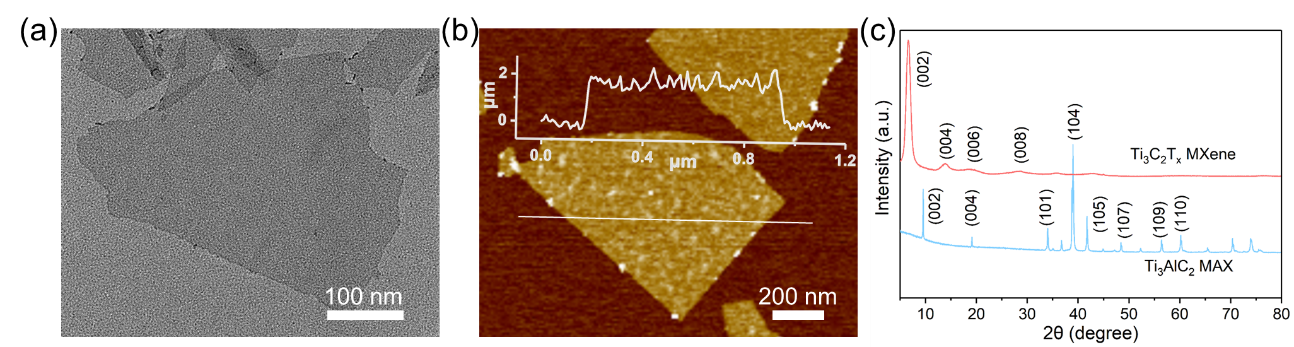


**Figure S2** Microstructure and structure characterization of Ti_3_C_2_T_x_ MXene nanosheets. (a) TEM. (b) AFM. (c) XRD.


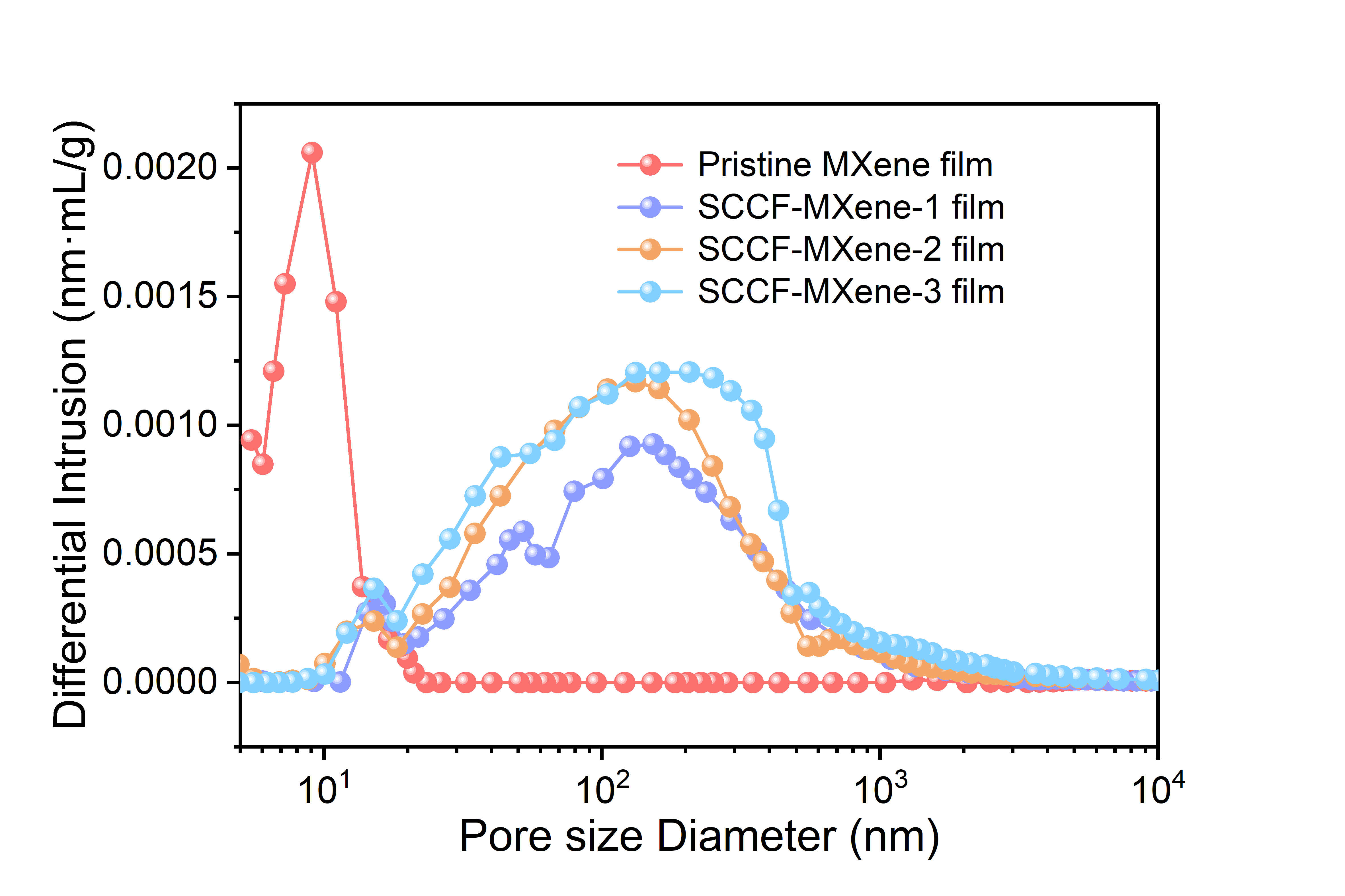


**Figure S3** Pore size distribution of MXene film and SCCF-MXene film

**Table S1** Average pore diameter and porosity

|  | Average pore diameter/nm | Porosity |
| --- | --- | --- |
| MXene film | 14.6 | 2.8% |
| SCCF-MXene-1 film | 294.7 | 73.4% |
| SCCF-MXene-2 film | 376.8 | 88.2% |
| SCCF-MXene-3 film | 389.3 | 88.5% |


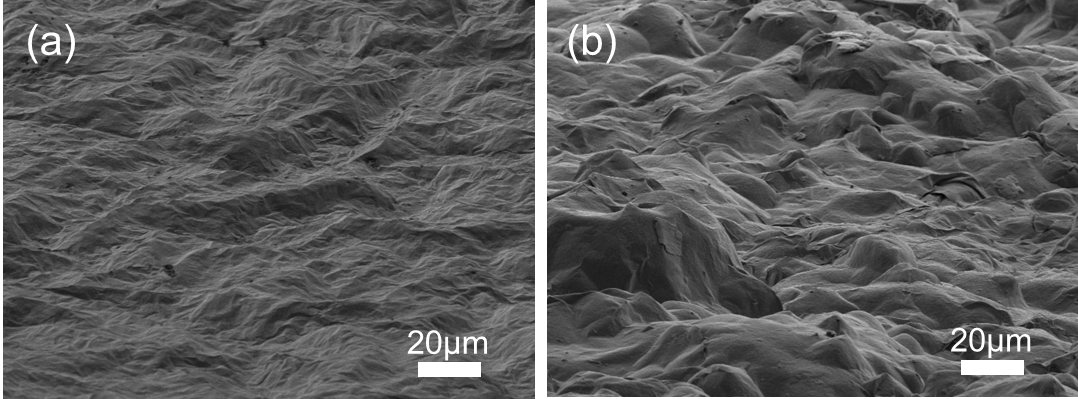


**Figure S4** Surface SEM of (a) MXene film and (b) SCCF-MXene film.


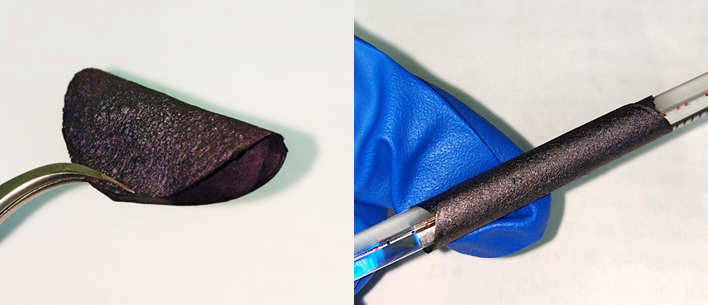


**Figure S5** Digital images for the bending and twisting state of SCCF-MXene film.


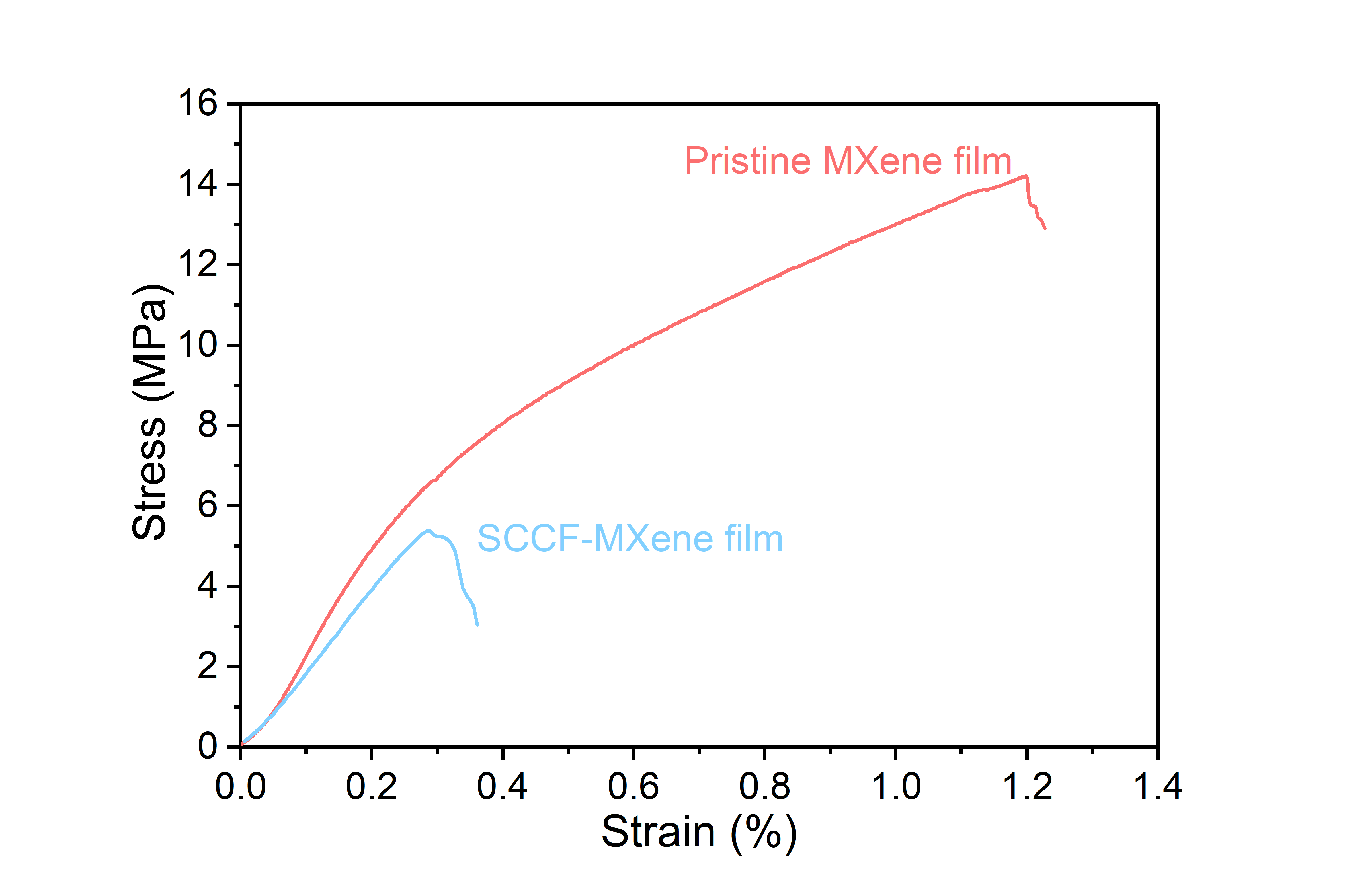


**Figure S6**. Tensile stress-strain curves of MXene film and SCCF-MXene film.


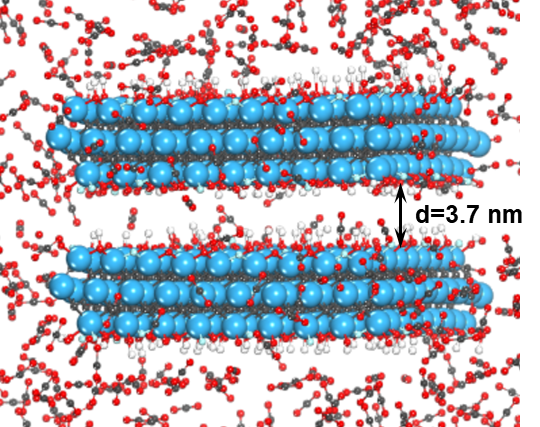


**Figure S7** Molecular dynamics simulations illustrating the MXene interlayer in supercritical CO_2_ environment of 0 MPa.


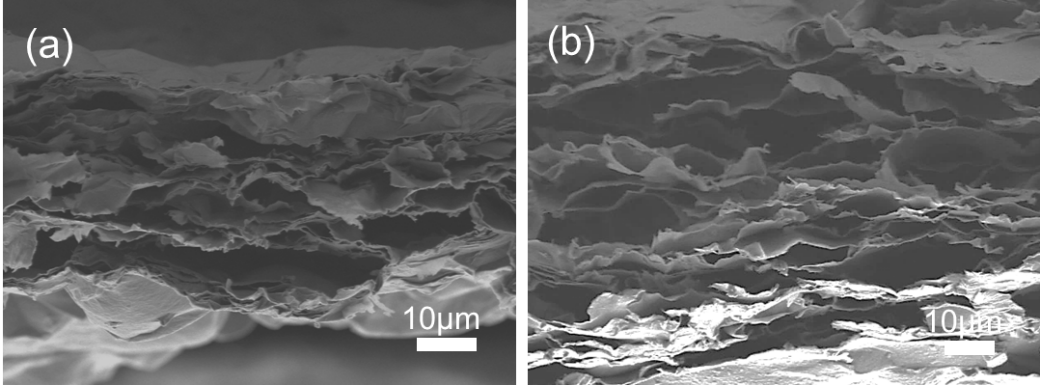


**Figure S8** Cross-sectional SEM images of (a) SCCF-MXene-1 film and (b) SCCF-MXene-3 film


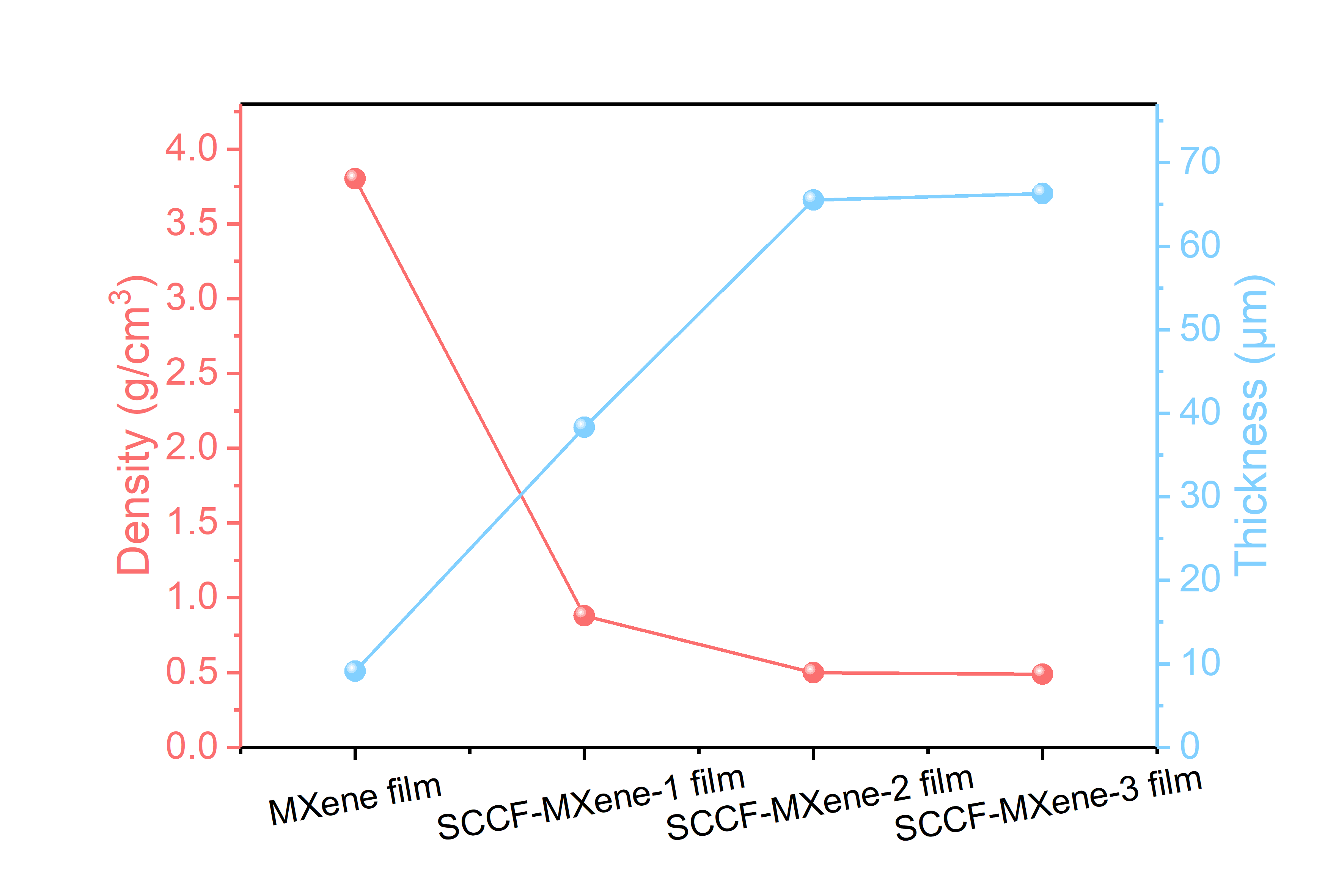


**Figure S9** Density and thickness of MXene and porous SCCF-MXene film


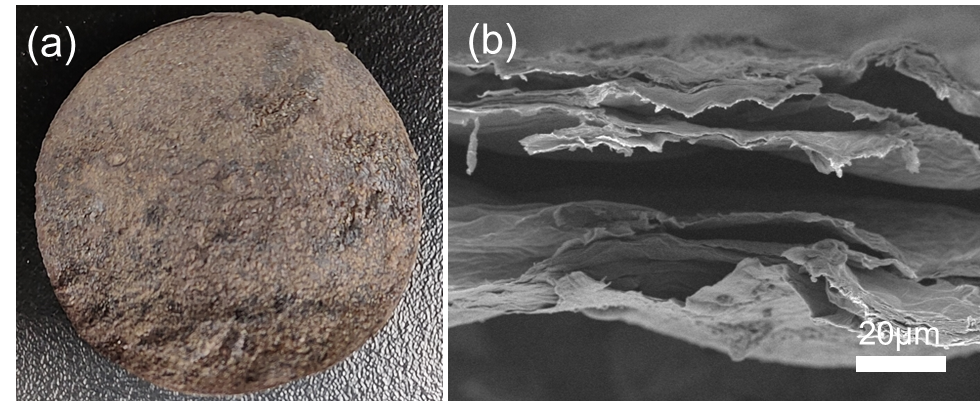


**Figure S10** Supercritical foaming of GO films


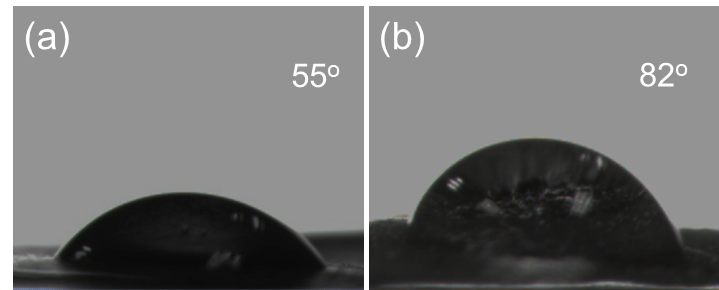


**Figure S11** Water contact angle of (a) MXene film and (b) SCCF-MXene film


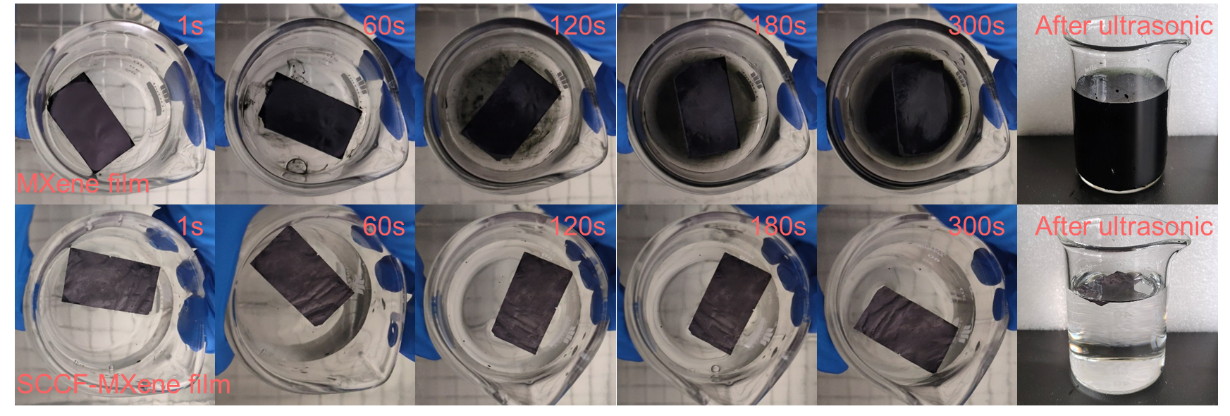


**Figure S12** The MXene film (top) and the SCCF-MXene film (bottom) being immersed in water under sonication for various times.


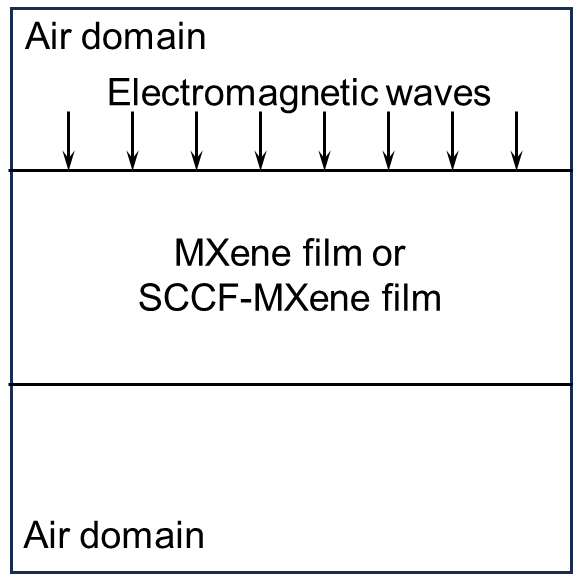


**Figure S13** Schematic diagram of the COMSOL simulation model.


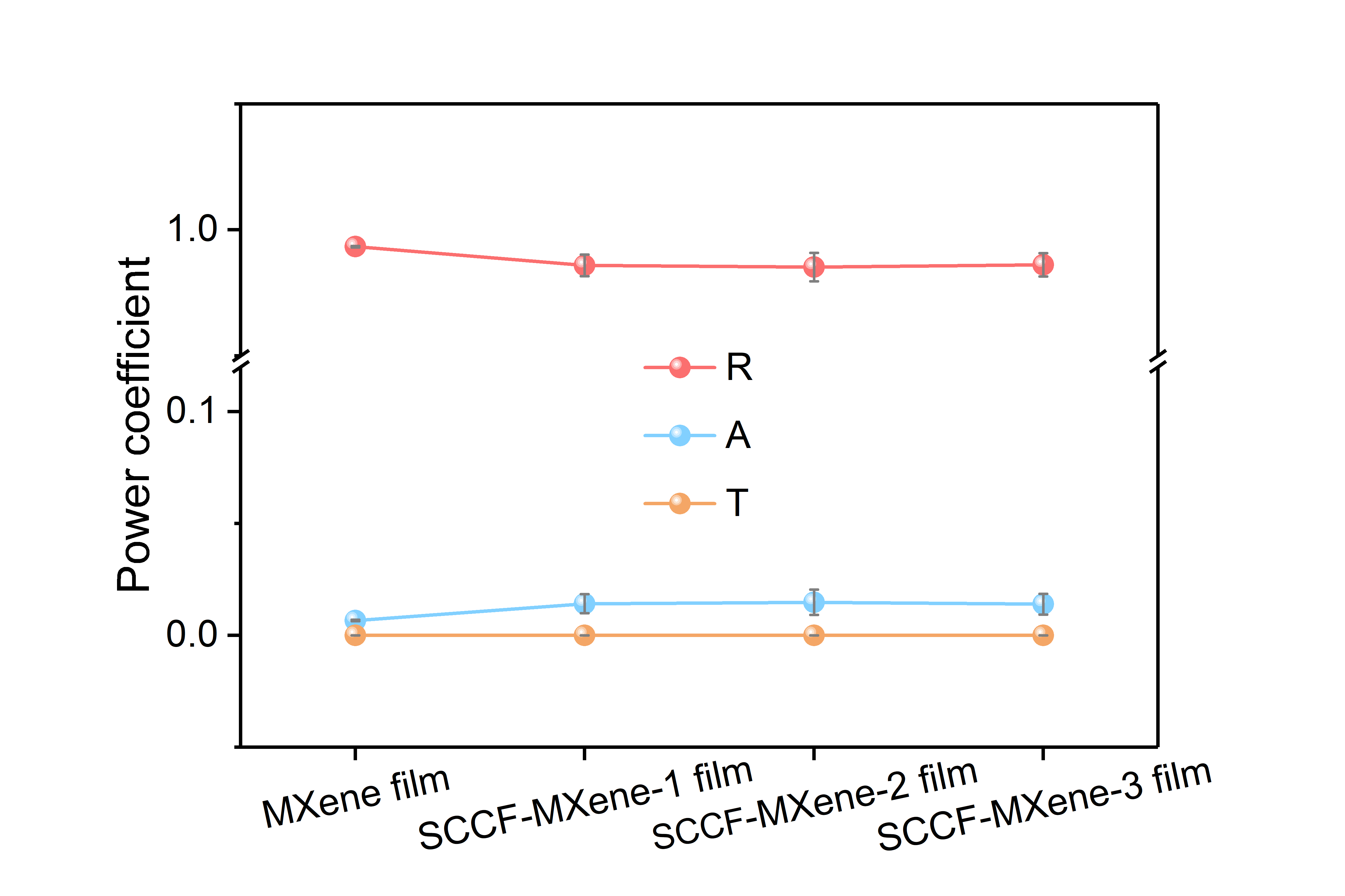


**Figure S14** Power coefficient of MXene and SCCF-MXene film.


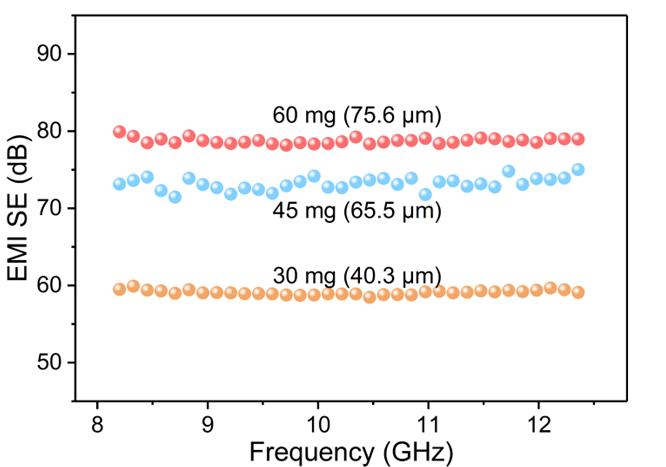


**Figure S15** EMI SE of SCCF-MXene-2 film at various thickness.


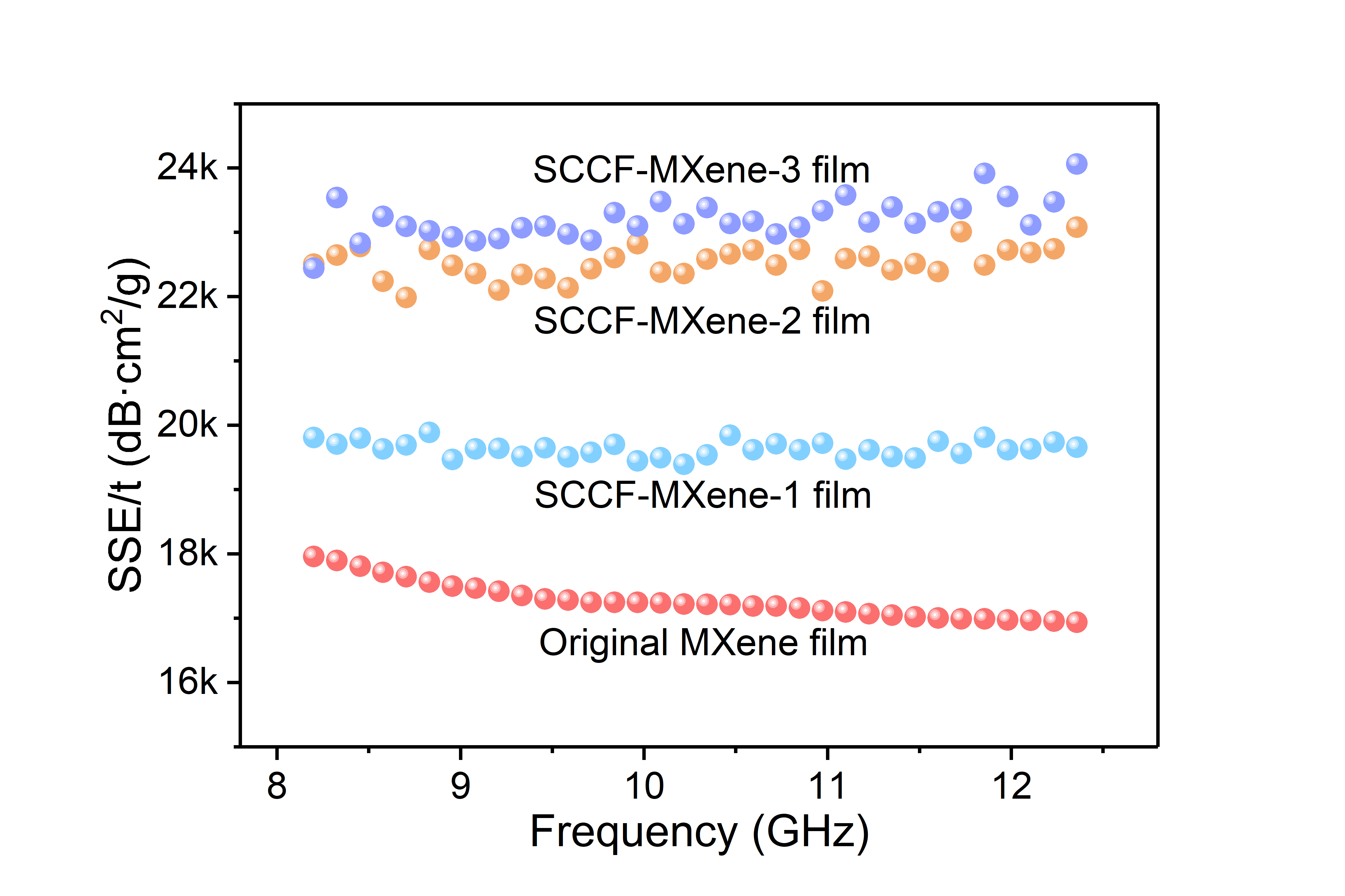


**Figure S16** SSE/t of the MXene film and SCCF-MXene film.


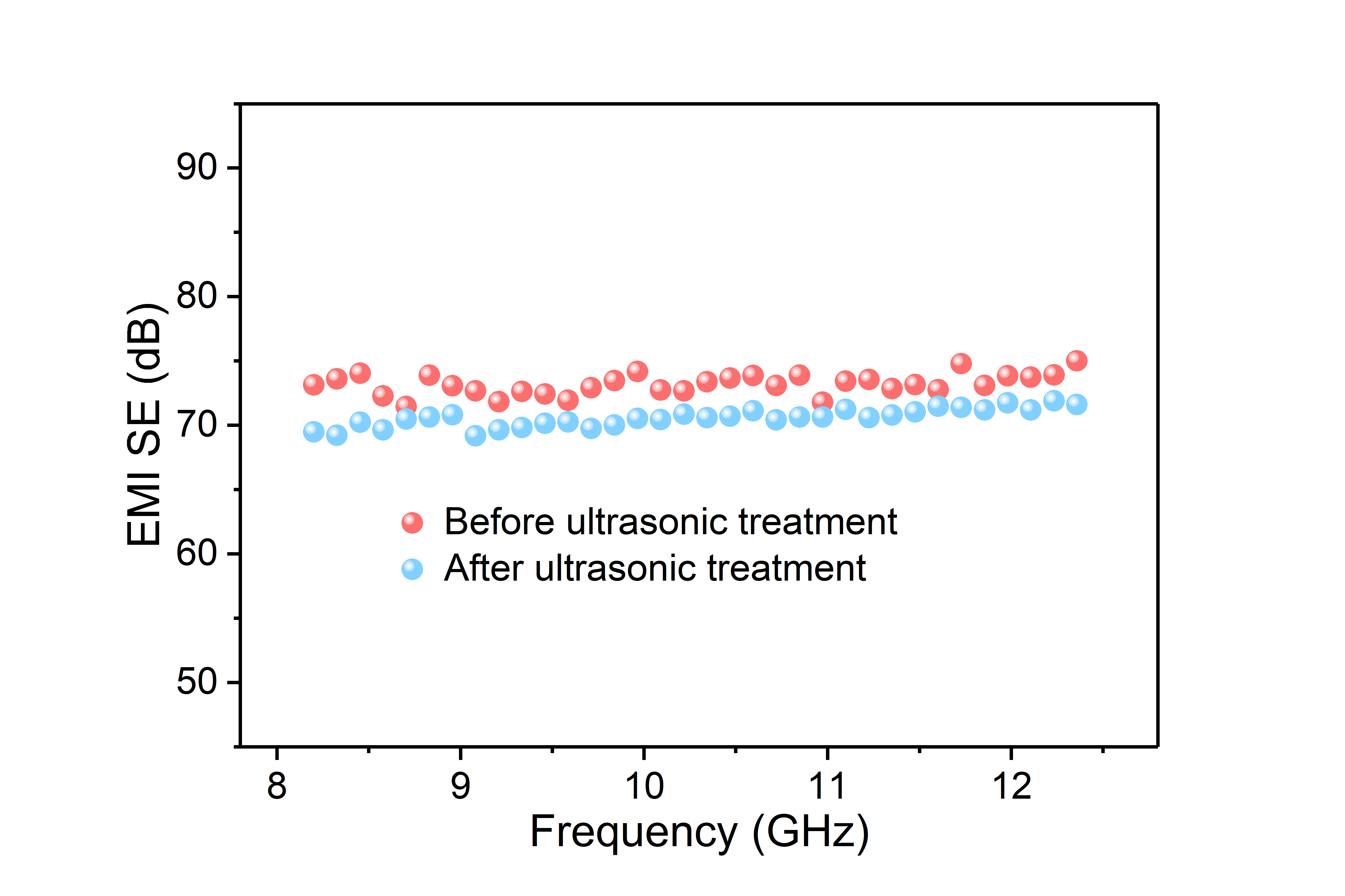


**Figure S17** EMI SE of SCCF-MXene films before and after sonication for 1 h.


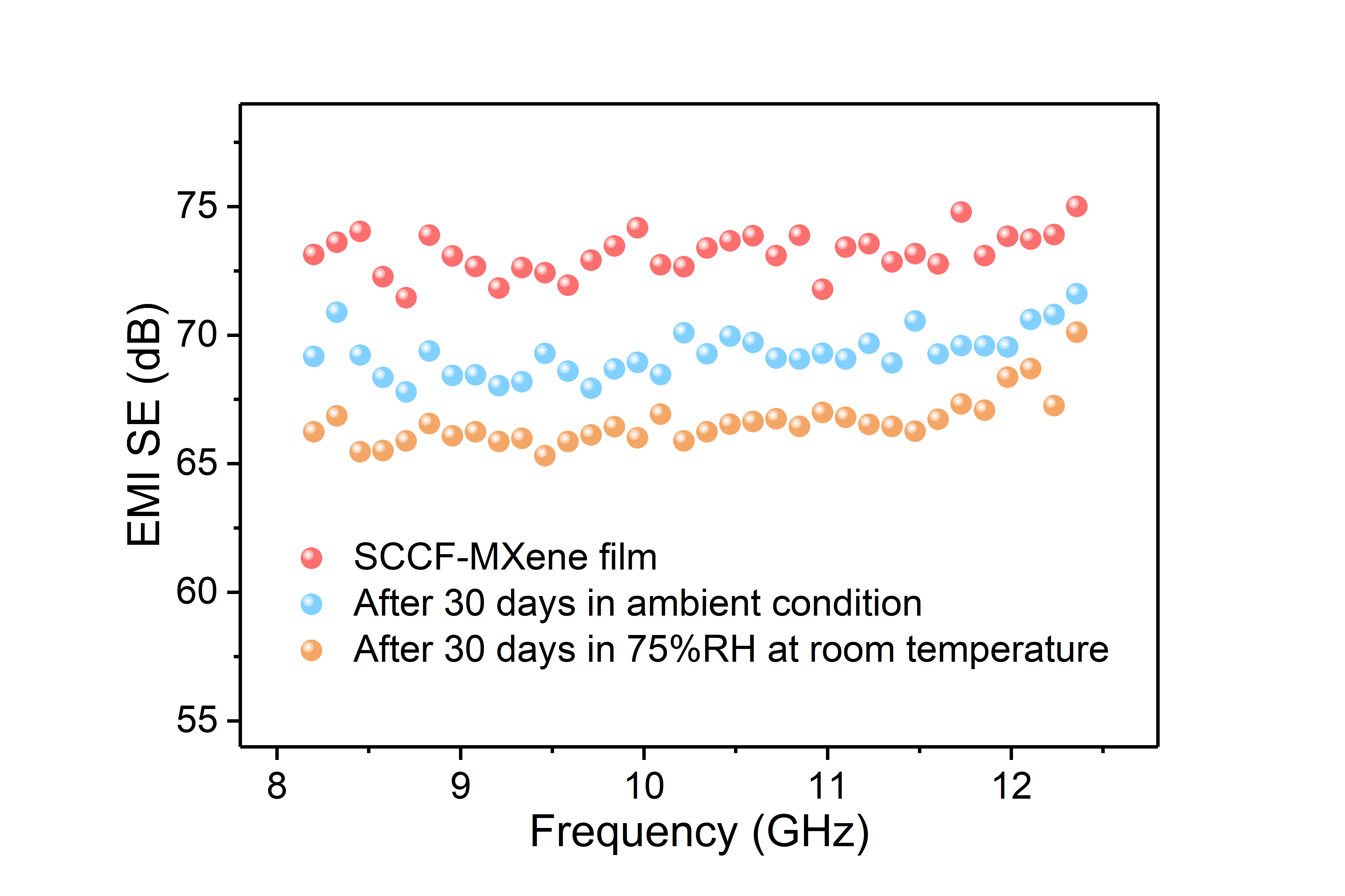


**Figure S18** EMI SE of SCCF-MXene films after being stored at the ambient conditions and 75%RH at room temperature for 30 days.


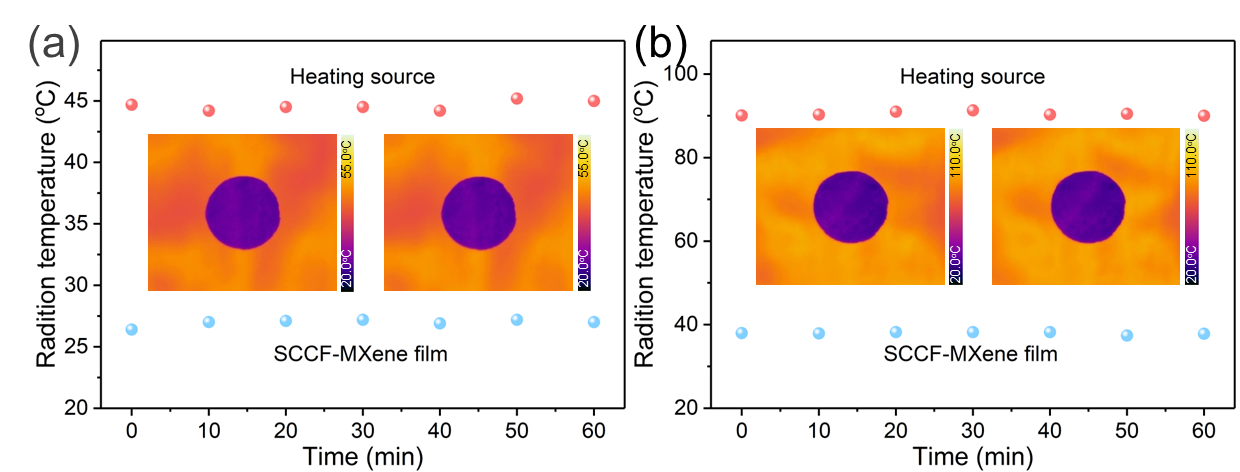


**Figure S19** Infrared stealth performance of SCCF-MXene film continuously placed on a heating stage at (a) 45^o^C and (b) 90^o^C


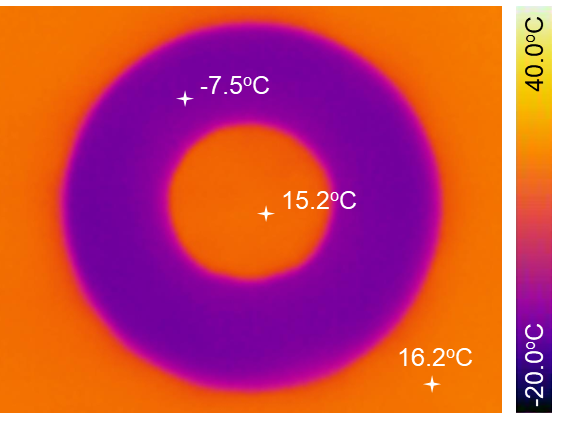


**Figure S20** Infrared image of SCCF-MXene film applied in objects below ambient temperature.

**Table S2** Comparison of SSE/*t* and *t* of SCCF-MXene films and the reported MXene-based EMI shielding materials

| Materials | EMI SE (dB) | *t*  (mm) | *ρ* (g/cm^3^) | SSE/*t*  (dB cm^2^/g) | Ref. |
| --- | --- | --- | --- | --- | --- |
| **SCCF-MXene film** | **73.2** | **0.065** | **0.5** | **22523** | ***This work*** |
|  | **74.9** | **0.066** | **0.49** | **23160** |  |
| MXene foam | 70 | 0.06 | 0.22 | 53030 | 1 |
| Reconstructed MXene film | 73 | ~0.275 | 0.11 | 25298 | 2 |
| NF-MXene | 73 | 0.04 | 0.72 | 25347 | 3 |
| 3D MXene film | 75 | 0.06 | 0.6 | 20833 | 4 |
| Hollow MXene film | 69.2 | 0.05 | 0.59 | 23458 | 5 |
| Heat treated Ti_3_CNT_x_ foam | 118.4 | 0.376 | ~0.11 | 27858 | 6 |
| HM treated Ti_3_CNT_x_ foam | 74.4 | 0.312 | ~0.17 | 14108 |  |
| Ti_3_CNT_x_ MXene aerogel | 54.1 | 1 | 0.011 | 49182 | 7 |
| 3D MXene Frame | 81.8 | 1.34 | 0.162 | 3768 | 8 |
| MXene/carbon foam | 74 | 0.3 | 0.34 | 7255 | 9 |
| HM treated rGO-MXene porous film | 52.6 | 0.13 | 0.263 | 15385 | 10 |
| Porous PI/MXene | 77.4 | 0.21 | 0.39 | 9451 | 11 |
| MXene/carbon foam | 25 | 2 | 0.005 | 25000 | 12 |
| Porous MXene/PI film | 62.5 | 1.5 | 0.0487 | 8556 | 13 |
| MXene/Ag NWs/melamine sponge | 24.9 | 2.05 | 0.012 | 10122 | 14 |
| PU@PDA/MXene foam | 72.2 | 2 | 0.289 | 1249 | 15 |
| MXene/ANF aerogel | 56.8 | 1.9 | 0.082 | 3646 | 16 |
| Cotton/MXene aerogel | 48.1 | 1.865 | 0.06 | 4298 | 17 |
| MXene/graphene aerogel | 75 | 1 | 0.03 | 25000 | 18 |
| MXene/CNTs/aramid aerogel | 69 | 2 | 0.428 | 806 | 19 |
| MXene/CNT hybrid aerogel | 103.9 | 3 | 0.042 | 8246 | 20 |
| MXene/rGO foam | 50.7 | 3 | 0.0046 | 36739 | 21 |
| MXene/wood-derived carbon aerogel | 71.3 | 3 | 0.197 | 1206 | 22 |

**Table S3** Comparison of through-plane thermal conductivity of SCCF-MXene film and previously reported MXene-based films.

| Materials | Thickness  (μm) | Through-plane thermal conductivity (W/(m·K)) | Ref. |
| --- | --- | --- | --- |
| **SCCF-MXene film** | **65** | **0.09** | This work |
| **Dense MXene film** | **18** | **0.21** |  |
| MXene film | 29 | 0.279 | 23 |
| MXene/MMT film | 25 | 0.27 | 24 |
| Hollow MXene film | 40 | 0.62 | 5 |
| MXene/CNF film | 21 | 0.52 | 25 |
| CNF/MXene/AgNWs film | 35 | 0.32 | 26 |
| MXene/CNF/LM film | 45 | 0.13 | 27 |
| MXene/PBO film | 10 | 0.103 | 28 |
| BP/MXene film | 30 | 0.37 | 29 |

**Reference:**

[1] J. Liu, H. Zhang, R. Sun, Y. Liu, Z. Liu, A. Zhou, Z. Yu. Hydrophobic, flexible, and lightweight MXene foams for high-performance electromagnetic-interference shielding. Advanced Materials 2017, 29(38), 1702367.

[2] X. Zhang, X. Liu, Q. Liu, Y. Feng, S. Qiu, T. Wang, H. Xu, H. Li, L. Yin, H. Kang, Z. Fan, Reversible constrained dissociation and reassembly of MXene films. Advanced Science 2024, 11, 2309171.

[3] L. Yin, Y. Yang, H. Yang, H. Kang, J. Wang, Y. Wang, Z. Xie, Y. Liu, Z. Fan, Rapid foaming of dense MXene films induced by acid-base neutralization reaction. Cell Reports Physical Science 2023, 4(6), 101421.

[4] L. Yin, H. Kang, H. Ma, J. Wang, Y. Liu, Z. Xie, Y. Wang, Z. Fan, Sunshine foaming of compact Ti_3_C_2_T_x_ MXene film for highly efficient electromagnetic interference shielding and energy storage. Carbon 2021, 182, 124-133.

[5] Y. Wang, C. Zhao, Y. Tian, Y. Sun, M. Zhang, K. Wang, B. Xia, Y. Wang, T. Li, X. Zhang, J. Huang, S. Wang, W. Dong, J. Qiao, Lightweight MXene composite films with hollow egg-box structures: enhanced electromagnetic shielding performance beyond pure MXene. Advanced Science 2025, 12, 2411932.

[6] R. Rahmati, M. Salari, M. Ashouri-Sanjani, A. Salehi, M. Hamidinejad, C. B. Park, Comparative effects of hydrazine and thermal reduction methods on electromagnetic interference shielding characteristics in foamed titanium carbonitride MXene films. Small 2024, 20, 2308320.

[7] M. Han, X. Yin, K. Hantanasirisakul, X. Li, A. Iqbal, C. B. Hatter, B. Anasori, C. M. Koo, T. Torita, Y. Soda, L. Zhang, L. Cheng, Y. Gogotsi, Anisotropic MXene aerogels with a mechanically tunable ratio of electromagnetic wave reflection to absorption. Advanced Optical Materials 2019, 7, 1900267.

[8] X. Wu, T.Tu, Y. Dai, P. Tang, Y. Zhang, Z. Deng, L. Li, H. Zhang, Z. Yu. Direct ink writing of highly conductive MXene frames for tunable electromagnetic interference shielding and electromagnetic wave-induced thermochromism. Nano-Micro Letters 2022, 13, 148.

[9] F. Qi, L. Wang, Y. Zhang, Z. Ma, H. Qiu, J. Gu, Robust Ti_3_C_2_T_x_ MXene/starch derived carbon foam composites for superior EMI shielding and thermal insulation. Materials Today Physics 2021, 21, 100512.

[10] Y. Zhang, M. Xu, Z. Wang, T. Zhao, L. Liu, H. Zhang Z. Yu. Strong and conductive reduced graphene oxide-MXene porous films for efficient electromagnetic interference shielding. Nano Research 2022, 15, 4916-492.

[11] Y. Cheng, X. Li, Y. Qin, Y. Fang, G. Liu, Z. Wang, J. Matz, P. Dong, J. Shen, M. Ye. Hierarchically porous polyimide/Ti_3_C_2_T_x_ film with stable electromagnetic interference shielding after resisting harsh conditions. Science Advances 2021, 7, eabj1663

[12] Y. Lu, X. Zhao, Y. Lin, P. Li, Y. Tao, Z. Wang, J. Ma, H. Xu, Y. Liu. Lightweight MXene/carbon composite foam with hollow skeleton for air-stable, high-temperature-resistant and compressible electromagnetic interference shielding. Carbon 2023, 206, 375-382.

[13] Z. Zeng, N. Wu, J. Wei, Y. Yang, T. Wu, B. Li, S.B. Hauser, W. Yang, J. Liu, S. Zhao. Porous and ultra-flexible crosslinked MXene/polyimide composites for multifunctional electromagnetic interference shielding. Nano-Micro Letters 2022, 14, 59.

[14] S. Wang, D. Li, W. Meng, L. Jiang, D. Fang. Scalable, superelastic, and superhydrophobic MXene/silver nanowire/melamine hybrid sponges for high-performance electromagnetic interference shielding. Journal of Materials Chemistry C, 2022,10, 5336-5344.

[15] Z. Li, Y. Sun, B. Zhou, Y. Feng, C. Liu, C. Shen, Flexible thermoplastic polyurethane/MXene foams for compressible electromagnetic interference shielding. Materials Today Physics 2023, 32, 101017.

[16] Z. Lu, F. Jia, L. Zhuo, D. Ning, K. Gao, F. Xie. Micro-porous MXene/aramid nanofibers hybrid aerogel with reversible compression and efficient EMI shielding performance. Composites Part B: Engineering 2021, 217, 108853.

[17] J. Zhai, C. Cui, A. Li, R. Guo, C. Cheng, E. Ren, H. Xiao, M. Zhou, J. Zhang. Waste cotton Fabric/MXene composite aerogel with heat generation and insulation for efficient electromagnetic interference shielding. Ceramics International 2022, 48, 13464-13474.

[18] T. Hua, H. Guo, J. Qin, Q. Wu, L. Li, B. Qian. 3D printing lamellar Ti_3_C_2_T_x_ MXene/graphene hybrid aerogels for enhanced electromagnetic interference shielding performance. RSC Advances, 2022, 12, 24980-24987.

[19] Z. Yan, Y. Ding, M. Huang, J. Li, Q. Han, M. Yang, W. Li. MXene/CNTs/Aramid aerogels for electromagnetic interference shielding and joule heating. ACS Applied Nano Materials 2023, 6, 6141-6150.

[20] P. Sambyal, A. Iqbal, J. Hong, H. Kim, M.K. Kim, S.M. Hong, M. Han, Y. Gogotsi, C.M. Koo. Ultralight and mechanically robust Ti_3_C_2_T_x_ hybrid aerogel reinforced by carbon nanotubes for electromagnetic interference shielding. ACS Applied Materials & Interfaces 2019 11 (41), 38046-38054.

[21] Z. Fan, D. Wang, Y. Yuan, Y. Wang, Z. Cheng, Y. Liu, Z. Xie. A lightweight and conductive MXene/graphene hybrid foam for superior electromagnetic interference shielding. Chemical Engineering Journal 2020, 381, 122696.

[22] C. Liang, H. Qiu, P. Song, X. Shi, J. Kong, J. Gu. Ultra-light MXene aerogel/wood-derived porous carbon composites with wall-like “mortar/brick” structures for electromagnetic interference shielding. Science Bulletin, 2020, 65, 616-622.

[23] L. Li, M. Shi, X. Liu, X. Jin, Y. Cao, Y. Yang, W. Wang, J. Wang, Ultrathin titanium carbide (MXene) films for high-temperature thermal camouflage. *Adv. Funct. Mater.* **2021**, *31*, 2101381

[24] L. Li, Y. Cao, X. Liu, J. Wang, Y. Yang, W. Wang, Multifunctional MXene-based fireproof electromagnetic shielding films with exceptional anisotropic heat dissipation capability and joule heating performance. *ACS Appl. Mater. Interfaces* **2020**, *12*, 27350-27360.

[25] G. Song, R. Kang, L. Guo, Z. Ali, X. Chen, Z. Zhang, C. Yan, C.T. Lin, N. Jiang, J. Yu, Highly flexible few-layer Ti_3_C_2_ MXene/cellulose nanofiber heat-spreader films with enhanced thermal conductivity. *New J. Chem.* **2020**, *44*, 7186-7193.

[26] B. Zhou, Q. Li, P. Xu, Y. Feng, J. Ma, C. Liu, C. Shen, An asymmetric sandwich structural cellulose-based film with self-supported MXene and AgNW layers for flexible electromagnetic interference shielding and thermal management. *Nanoscale* **2021**, *13*, 2378-2388.

[27] L. Ran, X. Ma, L. Qiu, F. Sun, L. Zhao, L. Yi, X. Ji, Liquid metal assisted fabrication of MXene-based films: Toward superior electromagnetic interference shielding and thermal management. *J. Colloid Interface Sci.* **2023**, *652*, 705-717.

[28] Y. Liu, W. Zou, N. Zhao, J. Xu, Electrically insulating PBO/MXene film with superior thermal conductivity, mechanical properties, thermal stability, and flame retardancy. *Nat. Commun.* **2023**, *14*, 5342.

[29] Y. Zhan, K. Wu, W. Xue, Facile preparation of MXene/BP nanocomposite films with high thermal conductivity and excellent flame retardancy. *Nanoscale Horiz.* **2025**, *10*, 3093.
